# Prognostic Biomarkers and Immunotherapeutic Targets Among CXC Chemokines in Pancreatic Adenocarcinoma

**DOI:** 10.3389/fonc.2021.711402

**Published:** 2021-08-23

**Authors:** Jiacheng Huang, Zhitao Chen, Chenchen Ding, Shengzhang Lin, Dalong Wan, Kuiwu Ren

**Affiliations:** ^1^Hepatobiliary and Pancreatic Surgery, Shulan (Hangzhou) Hospital Affiliated to Zhejiang Shuren University Shulan International Medical College, Hangzhou, China; ^2^School of Medicine, Zhejiang University, Hangzhou, China; ^3^First Affiliated Hospital, School of Medicine, Zhejiang University, Hangzhou, China; ^4^Fuyang People’s Hospital, Fuyang, China

**Keywords:** pancreatic cancer, CXC chemokines, prognostic biomarkers, immunotherapeutic targets, bioinformatics

## Abstract

**Background:**

Pancreatic cancer is one of the principal causes of tumor-related death worldwide. CXC chemokines, a subfamily of functional chemotactic peptides, affect the initiation of tumor cells and clinical outcomes in several human malignant tumors. However, the specific biological functions and clinical significance of CXC chemokines in pancreatic cancer have not been clarified.

**Methods:**

Bioinformatics analysis tools and databases, including ONCOMINE, GEPIA2, the Human Protein Atlas, DAVID, GeneMANIA, cBioPortal, STRING, DGidb, MethSurv, TRRUST, SurvExpress, SurvivalMeth, and TIMER, were utilized to clarify the clinical significance and biological functions of CXC chemokine in pancreatic cancer.

**Results:**

Except for CXCL11/12, the transcriptional levels of other CXC chemokines in PAAD tissues were significantly elevated, and the expression level of CXCL16 was the highest among these CXC chemokines. Our findings also suggested that all of the CXC chemokines were linked to tumor-immune dysfunction involving the abundance of immune cell infiltration, and the Cox proportional hazard model confirmed that dendritic and CXCL3/5/7/8/11/17 were significantly associated with the clinical outcome of PAAD patients. Furthermore, increasing expressions of CXCL5/9/10/11/17 were related to unfavorable overall survival (OS), and only CXCL17 was a prognostic factor for disease-free survival (DFS) in PAAD patients. The expression pattern and prognostic power of CXC chemokines were further validated in the independent GSE62452 dataset. For the prognostic value of single CpG of DNA methylation of CXC chemokines in patients with PAAD, we identified 3 CpGs of CXCL1, 2 CpGs of CXCL2, 2 CpGs of CXCL3, 3 CpGs of CXCL4, 10 CpGs of CXCL5, 1 CpG of CXCL6, 1 CpG of CXCL7, 3 CpGs of CXCL12, 3 CpGs of CXCL14, and 5 CpGs of CXCL17 that were significantly associated with prognosis in PAAD patients. Moreover, the prognostic value of CXC chemokine signature in PAAD was explored and tested in two independent cohort, and results indicated that the patients in the low-risk group had a better OS compared with the high-risk group. Survival analysis of the DNA methylation of CXC chemokine signature demonstrated that PAAD patients in the high-risk group had longer survival times.

**Conclusions:**

These findings reveal the novel insights into CXC chemokine expression and their biological functions in the pancreatic cancers, which might serve as accurate prognostic biomarkers and suitable immunotherapeutic targets for patients with pancreatic cancer.

## Introduction

Pancreatic cancer is one of the top five lethal malignant tumors, with an extremely poor prognosis (1, 2). Pancreatic adenocarcinoma (PAAD) is the major histological subtype of primary pancreatic cancer, accounting for more than 85% of all pancreatic cancers (3, 4). Surgical resection is the only potentially curative treatment. However, the effect of surgical treatment remains inadequate for advanced patients (5). To overcome this disease, other therapies such as radiation, immunotherapy, targeted therapy, and adjuvant chemotherapy are under research (6, 7). Targeted therapy and immunotherapy are emerging novel methods for pancreatic cancer therapy. Unfortunately, the complex relationship between the tumor microenvironment (TME), therapeutic targets, and the tumor-related immune dysfunction affects the multimodality management of PAAD patients (5, 8). Recently, many researchers have explored the therapeutic targets and immune checkpoint inhibitors of pancreatic cancer, and some progress has been made (2, 9). However, this is far from sufficient, and more therapeutic targets and signaling pathway must be identified.

CXC chemokines (CXCL1 to 17), a subfamily of soluble cytokines with small and highly conserved cytokines, are mainly concentrated in the TME and play an essential role in the initiation and progression of various tumors by binding to G-protein-coupled receptors (GPCRs) (10, 11). To date, six receptors for the CXC chemokines have been identified in humans (CXCR1 to 6). The TME, including malignant cells, immune cells, vasculature, extracellular matrix (ECM), tumor-associated endothelial cells, and tumor-associated fibroblasts, has profound effects on therapeutic response and clinical outcome through the interaction between circulatory, lymphatic systems, and surrounding cells (12, 13). Previous studies have identified that the TME plays a crucial role in microenvironment-mediated drug resistance (13). Therefore, modifying the factors and cells in the TME during the management of malignancies can improve the effectiveness of drug treatment (13). As an essential subunit of the chemokine family, the CXC chemokines can modulate the TME and biological phenotypes, affecting tumorigenesis, tumor-related therapeutic effect, tumor metastasis, and patient outcomes (14). Moreover, within the TME, CXC chemokine expression is often altered compared to normal tissue. However, to date, tumor-related immune cell infiltration and the interaction between the biological characteristics and clinical significance among CXC chemokines in PAAD remain unclear.

In this study, comprehensive bioinformatics was performed and analyzed for the CXC chemokines in patients with pancreatic cancer, and the biological characteristics, accurate prognostic biomarkers, and potential drug therapeutic targets were explored, helping further understand the molecular mechanism and management of pancreatic cancer.

## Materials and Methods

### Differential Expression, Transcriptional Levels, and Protein Expression

The differentially expressed CXC chemokines and its transcription levels in pancreatic cancer were identified through the ONCOMINE (15) (www.oncomine.org) and GEPIA2 (16) (http://gepia2.cancer-pku.cn/#index) bioinformatics analysis tools. In ONCOMINE database, the CXC chemokines satisfying statistically significant expression criteria *p*-value < 0.05 were screened, which ranked within the top 10% with fold change (FC) > 2. In GEPIA2 database, the *p-*value < 0.01 and |logFC | ≥ 1 were the cutoff criteria. Besides, the relative transcriptional levels of CXC chemokines were obtained in the GEPIA2 database, and the most highly expressed CXC chemokine was identified. Finally, the protein expression of the CXC chemokines family between pancreatic cancer and normal tissues were explored using the Human Protein Atlas database (https://www.proteinatlas.org/Version 20.1, updated: February 21, 2021).

### Functional and Pathway Enrichment Analysis

The GO (Gene Ontology) function analysis and KEGG (Kyoto Encyclopedia of Genes and Genomes) pathway enrichment analysis for CXC chemokines were conducted using DAVID (17, 18) (version 6.8, https://david.ncifcrf.gov/summary.jsp) and Metascape (https://metascape.org/). False discovery rate (FDR) < 0.05 and gene counts ≥ 10 were significant criteria.

### Immune Infiltration Analysis and Transcription Factor Targets

TIMER database (19) (version 10.5; https://cistrome.shinyapps.io/timer/) is a bioinformatics analysis online tool to explore and analyze tumor-infiltrating immune cells. TRRUST (20) (https://www.grnpedia.org/trrust/), an online analysis tool for the prediction of transcription factor targets, was utilized to understand the biological significance of CXC chemokines.

### Genetic Alteration, Survival Curves, Co-Expression, PPI Network, and Interaction Analyses

To fully understand the molecular features and biological characteristics of CXC chemokines, CXCL1–17 (not including CXCL15) were uploaded to the cBioportal (21) (www.cbioportal.org) database, STRING (22) (https://string-db.org/) database, and GeneMANIA (23) (http://www.genemania.org) database separately. cBioPortal was utilized to explore the information of genetic alteration, survival curves, and co-expression of CXC chemokines in PAAD. STRING, a significant online database for the comprehensive protein–protein interaction (PPI) analysis, was utilized to study how proteins interact with each other, and 0.4 was set as the minimum required interaction score. Finally, the interaction analyses of the CXC chemokines in PAAD were further explored by the GeneMANIA.

### Pathological Stages and Drug–Gene Interactions

To further verify the clinical significance of the CXC chemokines in PAAD patients, the expression levels at different pathological stages were all studied using the GEPIA2 database, an online bioinformatics analysis tool data from the TCGA database. DGidb (24) (https://dgidb.genome.wustl.edu/), a web server for discovering drug–gene interactions or potentially available drug categories, was used to explore the potential druggable genes and drugs of CXC chemokines in PAAD patients, and interaction score > 1 was the significant criterion.

### Survival Analysis

To assess whether CXC chemokines are accurate prognostic markers for predicting PAAD patient survival, we explored the prognostic value of a single CXC chemokine in survival of patients with PAAD based on GEPIA2, the statistically significant criteria considered on the basis Pr (>F) < 0.05. Meanwhile, the prognostic value of single DNA methylation CpG of CXC chemokines in PAAD patients was also explored using MethSurv (25) (https://biit.cs.ut.ee/methsurv/). Furthermore, to obtain a more comprehensive expression level and prognostic value of the CXC chemokine signature in pancreatic cancer, in this study, the 16 candidate CXC chemokines were used to calculate the risk score for each patient based on SurvExpress database (26) (Interface v2.0, Database Update April 9, 2021, http://bioinformatica.mty.itesm.mx:8080/Biomatec/SurvivaX.jsp). The computational formula was as follows: $\text{Risk}\;\text{score}=\Sigma_{\text{i}=1}^{\text{n}}\text{Beta}(\text{i})\;\times \;\text{x}(\text{i}),$ where x(i) and Beta (i) represent the expression level of each CXC chemokine and Beta based on multivariate Cox regression analysis (**Supplementary Table S1**), respectively. Subsequently, the corresponding PAAD patients were categorized into low-risk groups (*n* = 88) and high-risk groups (*n* = 88) based on the mean value of the risk score. Survival curve was explored according to the high- and low-risk groups of the CXC chemokines. Meanwhile, the prognostic value of the DNA methylation of CXC chemokine signature in patients with pancreatic cancer was also explored using SurvivalMeth (27) (http://bio-bigdata.hrbmu.edu.cn/survivalmeth/).

### External Validation of the Expression Pattern and Prognostic Power of CXC Chemokines

GSE62452 (28) based on the gene detection platform of GPL6244 [(HuGene-1_0-st)] Affymetrix Human Gene 1.0 ST Array [transcript (gene) version)] was downloaded from the Gene Expression Omnibus (GEO) online database. The 130 samples and corresponding follow-up information of pancreatic cancer samples were downloaded for further analysis. Next, the boxplot and Kaplan–Meier curve were utilized to test the expression pattern and prognostic value of each CXC chemokine, and *p* < 0.05 was the significant criterion.

## Results

### Differential Expression, Transcriptional Levels, and Protein Expression of CXC Chemokines in Patients With PAAD

**Figure 1:**
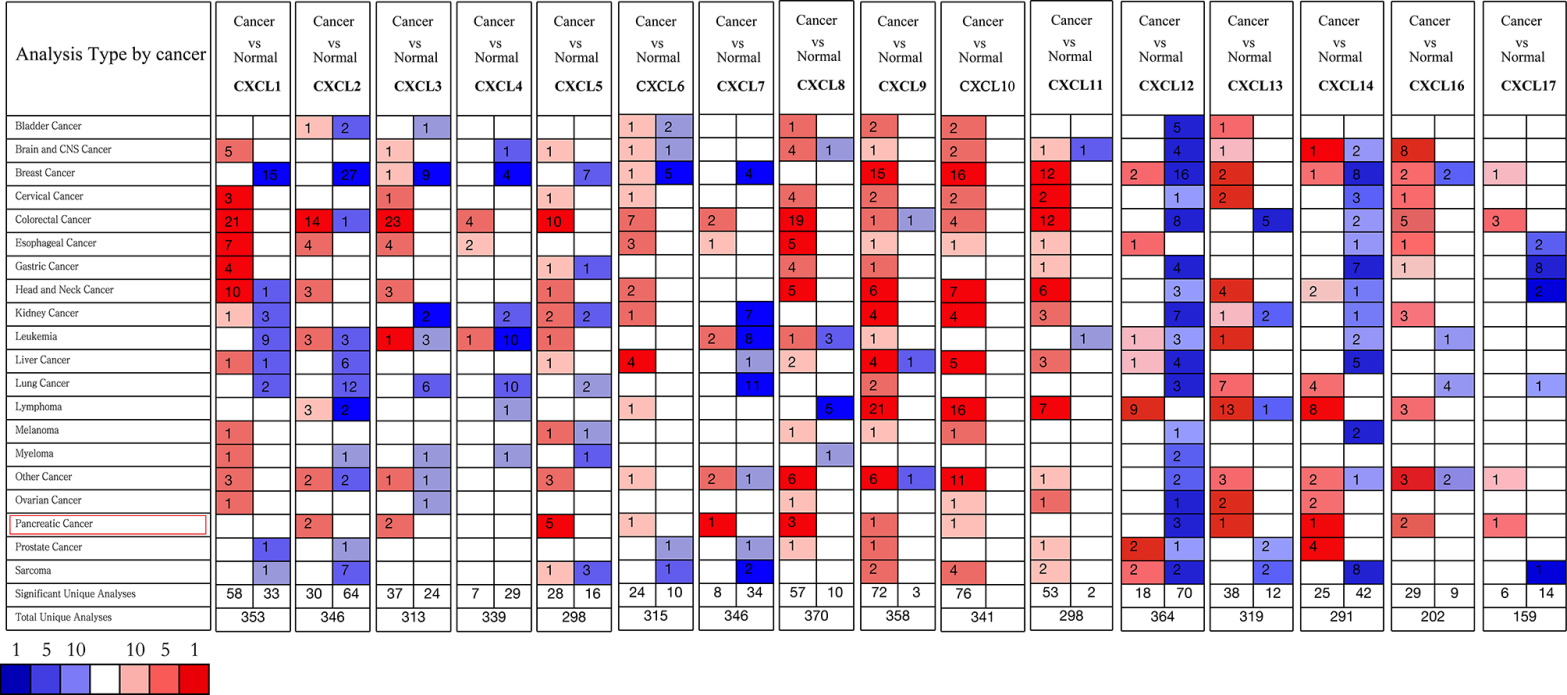
The transcriptional levels of CXC chemokines in several human malignant tumors (ONCOMINE). Upregulated expression (red); downregulated expression (blue).

**Figure 2:**
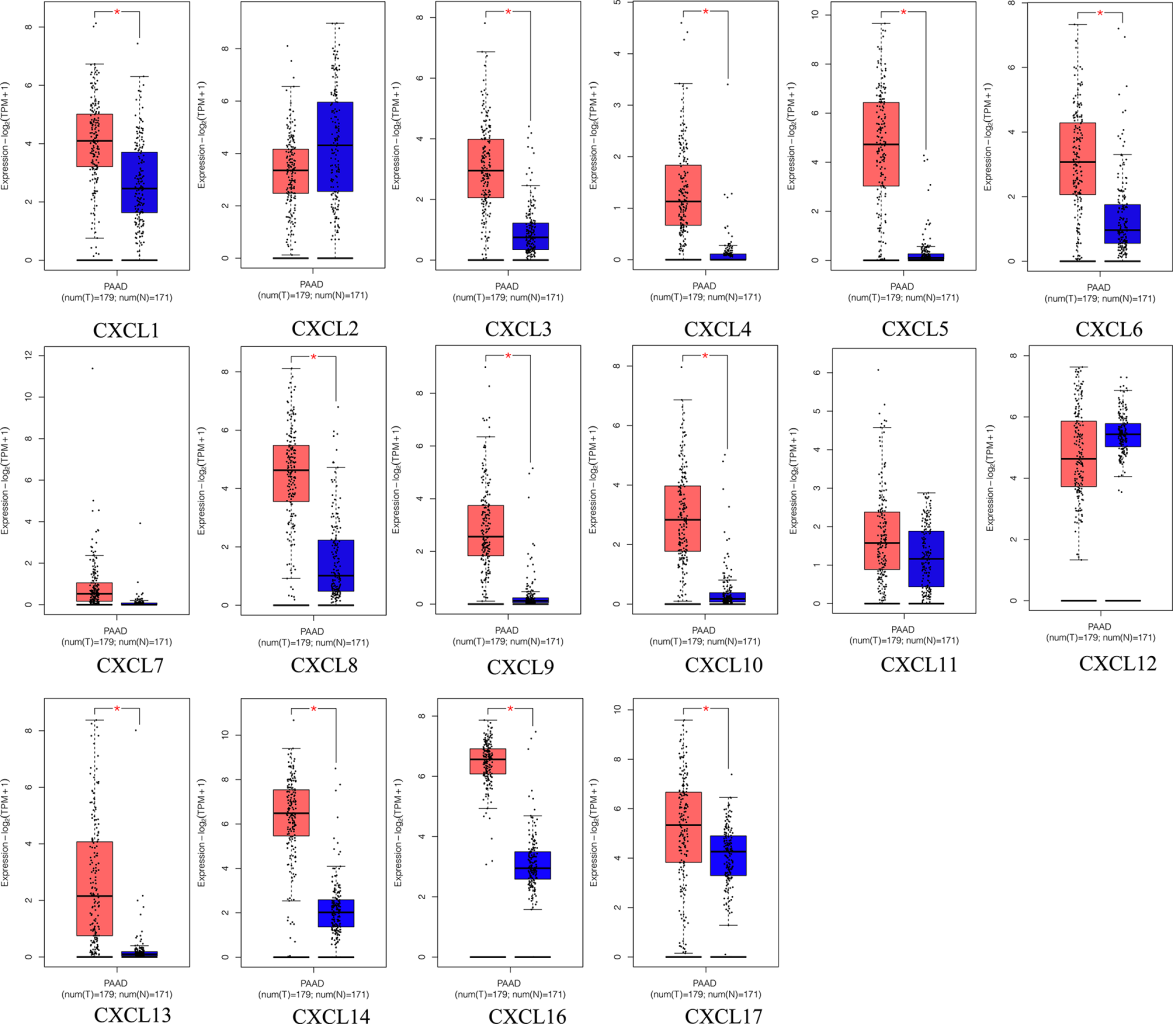
The transcriptional levels of diverse CXC chemokine family members in PAAD tissues and adjacent pancreatic tissues (GEPIA2). PAAD tissues (red); adjacent pancreatic tissues (blue). *p < 0.01 and |Log2FC| > 1.

**Figure 3:**
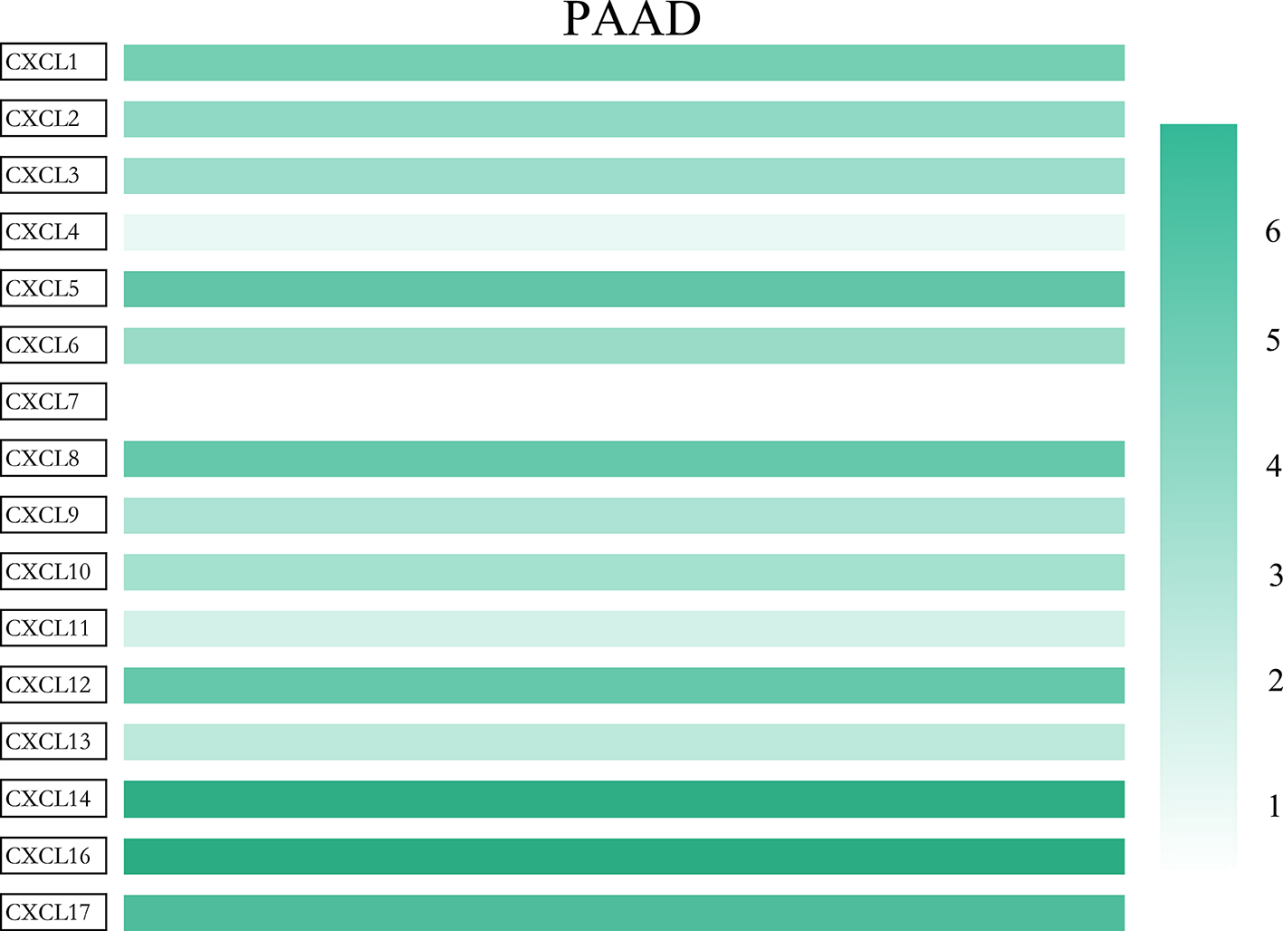
The relative expression level of CXC chemokine family members in LUAD (GEPIA2).

All the CXC chemokines (CXCL1 to 17) were explored using ONCOMINE. The expression levels in 16 CXC chemokines (not including CXCL15) between pancreatic cancer and normal pancreatic tissues were analyzed. Data from TCGA (the Cancer Genome Atlas database) revealed that the expression levels of CXCL2/3/5/6/7/8/9/10/13/14/16/17 were significantly higher in pancreatic cancer tissues than normal tissues (all *p*-value < 0.05, **Table 1** and **Figure 1**). Furthermore, differentially transcriptional levels of CXC chemokines in the GEPIA2 online database consistently showed higher transcriptional levels of CXCL1, CXCL3, CXCL4, CXCL5, CXCL6, CXCL8, CXCL9, CXCL10, CXCL13, CXCL14, CXCL16, and CXCL17 in PAAD (all *p* < 0.01, **Figure 2**). Thus, abnormally expressed CXC chemokines may serve as potential biomarker or immunotherapeutic targets in PAAD. The relative transcriptional levels of CXC chemokines in PAAD were compared through GEPIA2, and the result showed that the transcriptional level of CXCL16 was the highest, while that of CXCL7 was the lowest (**Figure 3**). Subsequently, we explored the protein expression of CXC chemokines family between pancreatic cancer and normal tissues using the Human Protein Atlas database (**Figure S1**). Unfortunately, none of the data assessed the protein expression of CXCL1/2/3/6/9/10/17 in pancreatic cancer and normal tissues.

**Table 1:** The transcriptional levels of abnormal expression CXC chemokines in different types of pancreatic cancer tissues (ONCOMINE).

Gene Symbol	Type	Fold Change	*p*-value	*t*-Test	Reporter	References
CXCL2	PAAD	2.632	0.047	2.351	IMAGE:380263	(30)
	PDAC	26.364	0.001	5.416	NM-002089	(31)
	PDAC	2.049	0.001	3.136	209774-x-at	(32)
CXCL3	PC	5.062	3.80E−7	5.806	207850-at	(33)
	PAAD	2.721	0.013	2.676	IMAGE:1556433	(30)
	PAAD	5.062	3.80E−7	5.806	207850-at	(32)
CXCL5	PC	12.881	5.58E−9	6.828	215101-s-at	(33)
	PDAC	13.978	3.37E−13	8.638	214974-x-at	(32)
	PAAD	5.697	6.38E−4	4.482	L37036-s-at	(34)
	PDAC	2.079	0.012	2.321	214974-x-at	(35)
	PC	4.451	8.20E−4	4.217	214974-x-at	(35)
CXCL6	PC	2.112	0.001	3.950	206336-at	(36)
	PAAD	2.410	0.012	2.984	IMAGE:2315207	(30)
	PDAC	4.393	1.85E−6	5.017	206336-at	(32)
CXCL7	PDAC	3.191	7.92E−5	4.139	214146-s-at	(35)
CXCL8	PC	4.573	1.71E−5	5.854	202859-x-at	(36)
	PDAC	9.800	9.92E−12	7.971	202859-x-at	(32)
	PC	8.378	3.51E−6	5.473	202859-x-at	(33)
	PAAD	6.984	0.039	2.221	Y00787-s-at	(34)
	PAAD	2.340	0.010	3.072	IMAGE:549933	(30)
CXCL9	PAAD	2.079	0.026	2.209	IMAGE:503617	(30)
	PDAC	2.377	6.21E−4	4.782	NM-002416	(31)
	PDAC	2.551	1.27E−5	4.490	203915-at	(32)
CXCL10	PC	3.950	1.41E−4	4.190	204533-at	(33)
CXCL13	PDAC	7.592	0.004	3.085	205242-at	(37)
	PDAC	2.455	0.010	2.398	205242-at	(32)
CXCL14	PAAD	12.119	1.49E−5	6.699	IMAGE:345034	(30)
	PC	4.326	0.004	3.250	218002-s-at	(36)
	PC	3.064	0.012	2.434	218002-s-at	(33)
	PDAC	3.915	1.51E−6	5.084	218002-s-at	(32)
CXCL16	PAAD	2.418	0.003	4.017	IMAGE:753278	(30)
	PC	2.022	4.05E−4	3.905	223454-at	(33)
	PDAC	2.311	6.64E−12	8.384	223454-at	(32)
CXCL17	PDCA	2.154	0.01	2.436	226960-at	(35)

CXCL, C-X-C chemokine ligand; PC, Pancreatic Carcinoma; PDAC, Pancreatic Ductal Adenocarcinoma; PAAD, pancreatic adenocarcinoma.

### Genetic Alteration, Survival Curves, Co-Expression, PPI Network, and Interaction Analyses of CXC Chemokines in Patients With PAAD

**Figure 4:**
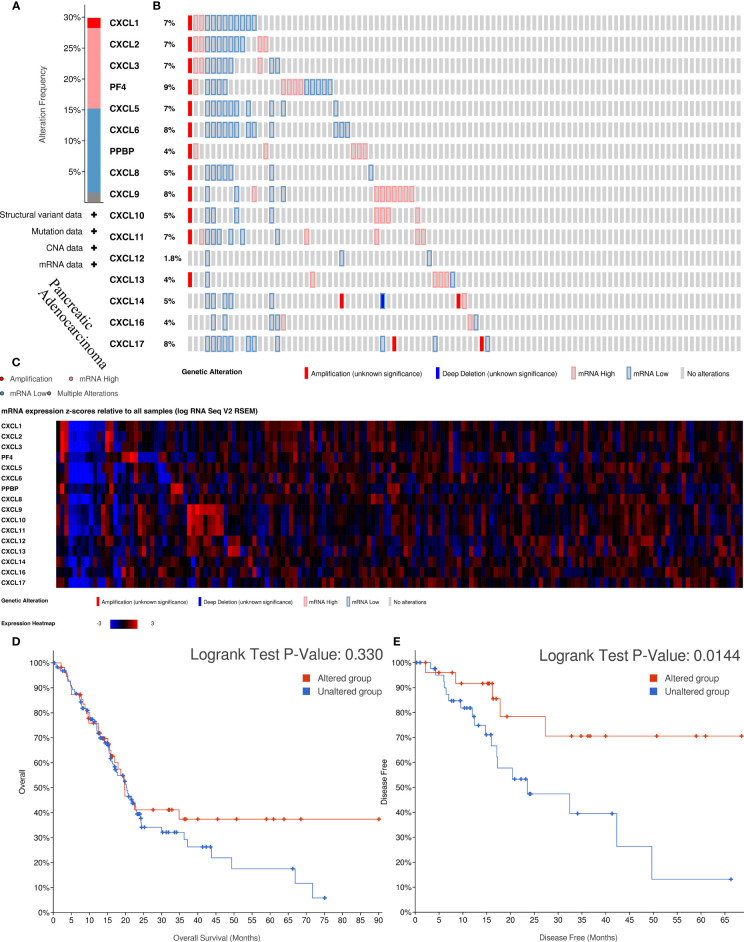
The biological functions and survival analysis of mutated CXC chemokines (cBioportal). **(A)** Summary of alterations in different expressed CXC chemokines family in PAAD. **(B)** Genetic alterations in CXC chemokines in PAAD. **(C)** The miRNA expression heatmap of CXC chemokines. **(D, E)** Survival curves of PAAD patients in altered and unaltered groups of the CXC chemokines. **(D)** OS; **(E)** DFS.

**Figure 5:**
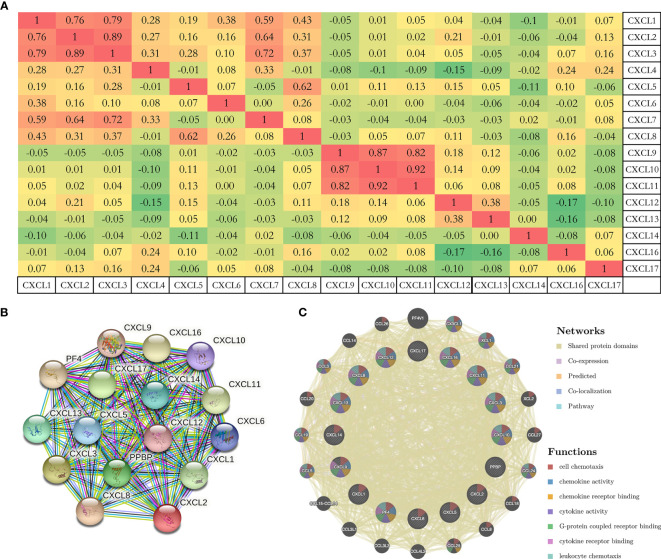
**(A)** The co-expression of CXC chemokines with each other based on the Pearson’s correlation coefficient for PAAD samples (cBioportal). **(B, C)** PPI network and functional relationship of CXC chemokines (STRING, GeneMANIA).

To further analyze the molecular characteristics and biologic functions of the CXC chemokines in PAAD, genetic alterations, survival curves, co-expression, PPI network, and biological interaction analyses of CXC chemokines in PAAD were performed. Firstly, the genetic alterations of CXC chemokines were carried out by the cBioPorta database. The results showed that CXC chemokines were altered in 29.89% of 184 cases (data from TCGA, PanCancer Atlas) (**Figure 4A**). In addition, CXCL1/2/3/4/5/6/7/8/9/10/11/12/13/14/16/17 were altered in 7%, 7%, 7%, 9%, 7%, 8%, 4%, 5%, 8%, 5%, 7%, 1.8%, 4%, 5%, 4%, and 8% of the sequencing data from PAAD samples, respectively (**Figure 4B**). Thus, CXCL4 is the highest mutated gene. Also, the miRNA expression heatmap of CXC chemokines was displayed (**Figure 4C**). Secondly, Kaplan–Meier curve results showed no noticeable discrepancy in overall survival (OS) between the altered group and the unaltered group (**Figure 4D**, *p* = 0.330). However, genetic alteration in CXC chemokines was associated with better disease-free survival (DFS) of PAAD patients (**Figure 4E**, *p* = 0.014). Thirdly, the co-expression of CXC chemokines was explored by the Pearson’s correlation coefficient for PAAD samples (TCGA, PanCancer Atlas), and the results showed a significantly positive correlation among the expression of CXCL1, CXCL2, CXCL3, CXCL9, CXCL10, and CXCL11 (**Figures 5A** and **S2**). BRCA1/2 mutated genes, first identified in breast cancer, are independent risk factors in the initiation and progression of several human malignant tumors (38, 39). Recent studies suggested that germline BRCA mutations are also correlated with an increased risk of developing pancreatic cancer, and up to 8% of patients with BRCA1/2 mutated genes (40). Several prospective clinical trials have demonstrated that DNA damage-related treatment based on BRCA gene mutation may be a safe and efficient treatment for BRCA1/2-deficient pancreatic cancers (40, 41). Subsequently, the co-expression relationship between CXC chemokines and BRCA1/2 gene mutation was evaluated using a scatter plot (**Figures S3** and **S4**). The results showed that BRCA1 gene mutation was significantly associated with CXCL7 (Pearson’s correlation = 0.21, *p* = 7.441e−3), CXCL9 (Pearson’s correlation = 0.28, *p* = 2.839e−4), CXCL10 (Pearson’s correlation = 0.36, *p* = 1.398e−6), and CXCL11 (Pearson’s correlation = 0.32, *p* = 2.031e−5). BRCA2 gene mutation was significantly associated with CXCL5 (Pearson’s correlation = 0.15, *p* = 0.0474), CXCL9 (Pearson’s correlation = 0.32, *p* = 1.810e−5), CXCL10 (Pearson’s correlation = 0.38, *p* = 5.05e−7), CXCL11 (Pearson’s correlation = 0.39, *p* = 2.01e−7), CXCL13 (Pearson’s correlation = 0.20, *p* = 0.0102), and CXCL 17 (Pearson’s correlation = −0.16, *p* = 0.0371). Fourth, a comprehensive PPI information network of the CXC chemokines was constructed using STRING. As expected, the network consisted of 16 nodes and 111 edges with the enrichment *p*-value < 1.0e−16 (**Figure 5B**). Finally, to further understand CXC chemokines, all CXC chemokines were uploaded to GeneMANIA to explore the significant relationship and functions. The results showed that the CXC chemokines markedly act on cell chemotaxis, chemokine receptor binding, chemokine activity, cytokine activity, G-protein-coupled receptor binding, cytokine receptor binding, and leukocyte chemotaxis (**Figure 5C**).

### Functional Enrichment Analyses of CXC Chemokines in Patients With PAAD

The GO (Gene Ontology) functional enrichment of CXC chemokines was analyzed using DAVID 6.8. As shown in **Table 2**, the CXC chemokines in the CC (cellular component) functional ontologies are mainly enriched in extracellular space and extracellular region. For the category of MF (molecular function), the CXC chemokines primarily cluster in chemokine activity, and the CXC chemokines in the BP (biological processes) functional ontologies are mainly enriched in chemotaxis, G-protein-coupled receptor signaling pathway, chemokine-mediated signaling pathway, inflammatory response, response to lipopolysaccharide, and immune response. In addition, the KEGG (Kyoto Encyclopedia of Genes and Genomes) pathway enrichment analysis shows that two typical pathways were overrepresented in CXC chemokines, including the chemokine signaling pathway and cytokine–cytokine receptor interaction. These results suggested that top significantly clustered GO function enrichment and KEGG pathways serve important biological roles during the initiation and progression of PAAD, providing new insights into the progression and potential immunotherapeutic targets for PAAD patients. Also, these results were confirmed in the Metascape for pathway and process enrichment analysis (**Figure S5** and **Table S2**).

**Table 2:** Significantly enriched GO terms and KEGG pathways of CXC chemokines in PAAD (DAVID 6.8).

Category	Term	Description	Count	FDR
GOTERM_CC_DIRECT	GO:0005615	Extracellular space	16	1.10E−16
GOTERM_CC_DIRECT	GO:0005576	Extracellular region	16	8.08E−16
GOTERM_MF_DIRECT	GO:0008009	Chemokine activity	15	8.14E−36
GOTERM_BP_DIRECT	GO:0006955	Immune response	14	5.02E−18
GOTERM_BP_DIRECT	GO:0070098	Chemokine-mediated signaling pathway	13	6.45E−25
GOTERM_BP_DIRECT	GO:0006954	Inflammatory response	13	1.83E−16
GOTERM_BP_DIRECT	GO:0007186	G-protein coupled receptor signaling pathway	13	2.96E−12
GOTERM_BP_DIRECT	GO:0006935	Chemotaxis	12	1.46E−19
GOTERM_BP_DIRECT	GO:0032496	Response to lipopolysaccharide	11	4.02E−16
KEGG_PATHWAY	hsa04062	Chemokine signaling pathway	11	2.79E−15
KEGG_PATHWAY	hsa04060	Cytokine-cytokine receptor interaction	11	2.15E−14

CXCL, C-X-C chemokine ligand; PAAD, pancreatic adenocarcinoma; CC, cellular component; MF, molecular function; BP, biological processes; GO, Gene Ontology; KEGG, Kyoto Encyclopedia of Genes and Genomes; FDR, false discovery rate.

### Immune Infiltration Analysis and Transcription Factor Targets of CXC Chemokines in Patients With PAAD

**Figure 6:**
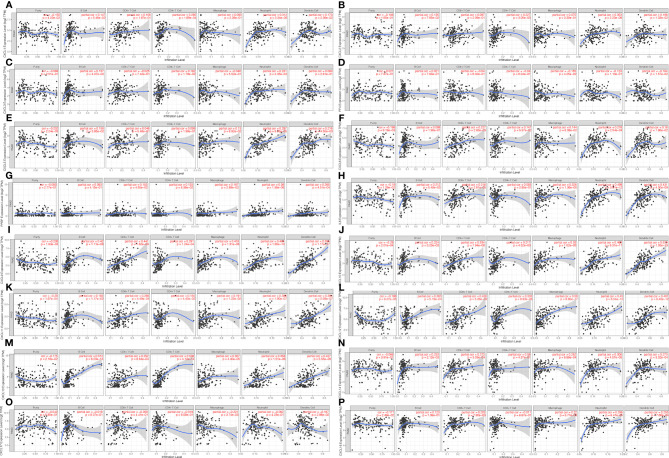
The correlation between CXC chemokines and immune cell infiltration (B cells, CD4+ T cells, CD8+ T cells, neutrophils, macrophages, and dendritic cells) in PAAD (TIMER). **(A)** CXCL1 **(B)** CXCL2 **(C)** CXCL3 **(D)** CXCL4 **(E)** CXCL5 **(F)** CXCL6 **(G)** CXCL7 **(H)** CXCL8 **(I)** CXCL9 **(J)** CXCL10 **(K)** CXCL11 **(L)** CXCL12 **(M)** CXCL13 **(N)** CXCL14 **(P)** CXCL16 **(O)** CXCL17.

All CXC chemokines were actively involved in immune cell infiltration and tumor-related inflammatory responses, thus affecting the tumorigenesis and tumor cell proliferation of PAAD. Results of **Figures 6A, B** show that the CXCL1 and CXCL2 are positively related to the infiltration of neutrophils, CD4+ T cells, and dendritic cells (*p* < 0.05). In addition, the findings showed that CXCL4 and CXCL17 are negatively correlated with the infiltration of macrophage and dendritic cells (*p* < 0.05, **Figures 6D, O**). Moreover, the CXCL7 and CXCL16 are positively correlated with the infiltration of CD8+ T cells, neutrophils, and dendritic cell macrophage (all *p* < 0.05, **Figures 6G, P**). There is a positive relationship between CXCL3 and the immune cell infiltration, including CD4+ T cells, neutrophils, and B cells (*p* < 0.05, **Figure 6C**). The immune cell infiltration of CXCL5 in B cells, neutrophils, and dendritic cells (p < 0.05, **Figure 6E**), CXCL6 in CD8+ T cells, neutrophils, and dendritic cell (p < 0.05, **Figure 6F**), CXCL6 in CD8+ T cells, neutrophils, and dendritic cell (p < 0.05, **Figure 6F**), CXCL8 and CXCL14 in B cells, CD8+ T cells, macrophage, neutrophils, and dendritic cell (p < 0.05, **Figures 6H, N**) is observed. CXCL9, CXCL10, CXCL11, CXCL12, and CXCL13 are positively related to the immune cell infiltration (*p* < 0.05, **Figures 6I–M**). Next, we compared the tumor infiltration levels among PAAD with different somatic copy number alterations for each CXC chemokine and found that the somatic copy number alterations for the CXC chemokines can inhibit CD4+ T cell infiltration (**Figure S6**). Furthermore, the Cox proportional hazard model confirmed that dendritic (*p* = 0.007), CXCL3 (*p* = 0.014), CXCL5 (*p* = 0.025), CXCL7 (*p* = 0.029), CXCL8 (*p* = 0.025), CXCL11 (*p* = 0.041), and CXCL17 (*p* = 0.011) were significantly associated with the clinical outcome of PAAD patients (**Table 3**).

**Table 3:** The Cox proportional hazard model of CXC chemokines and six tumor-infiltrating immune cells in PAAD (TIMER).

	Coef	HR	95%CI_l	95%CI_u	*p*-value	sig
**B_cell**	5.795	328.675	0.514	2.10E + 05	0.079	·
**CD8_Tcell**	2.993	19.945	0.018	2.24E + 04	0.404	
**CD4_Tcell**	−7.371	0.001	0.000	4.46E + 00	0.103	
**Macrophage**	−5.691	0.003	0.000	6.94E + 00	0.144	
**Neutrophil**	13.404	662821.961	0.005	8.45E + 13	0.159	
**Dendritic**	−5.988	0.003	0.000	1.93E -01	0.007	**
**CXCL1**	−0.312	0.732	0.494	1.09E + 00	0.120	
**CXCL2**	0.163	1.177	0.827	1.68E + 00	0.364	
**CXCL3**	−0.53	0.588	0.385	9.00E - 01	0.014	*
**CXCL4**	0.313	1.368	0.997	1.88E + 00	0.052	·
**CXCL5**	0.17	1.185	1.021	1.38E + 00	0.025	*
**CXCL6**	0.02	1.02	0.822	1.27E + 00	0.856	
**CXCL7**	0.205	1.227	1.021	1.48E + 00	0.029	*
**CXCL8**	0.327	1.387	1.042	1.85E + 00	0.025	*
**CXCL9**	0.126	1.134	0.820	1.57E + 00	0.447	
**CXCL10**	−0.097	0.907	0.588	1.40E + 00	0.66	
**CXCL11**	0.437	1.548	1.019	2.35E + 00	0.041	*
**CXCL12**	−0.003	0.997	0.773	1.29E + 00	0.980	
**CXCL13**	−0.012	0.988	0.829	1.18E + 00	0.890	
**CXCL14**	−0.049	0.952	0.815	1.11E + 00	0.539	
**CXCL16**	−0.236	0.789	0.501	1.24E + 00	0.308	
**CXCL17**	0.168	1.182	1.039	1.35E + 00	0.011	*

CXCL, C-X-C chemokine ligand; PAAD, pancreatic adenocarcinoma; HR, Hazard ratio; CI, confidence interval; sig, significance.

*p < 0.05.

**p < 0.01.

Subsequently, TRRUST was performed to investigate the potential transcription factor targets of CXC chemokines. The results showed that three key transcription factors, namely, NFKB1, RELA, and SP1, played important biological roles in the regulation of CXC chemokines. The key transcription factors of CXCL1, CXCL10, CXCL2, CXCL5, CXCL12, and CXCL8 were NFKB1 and RELA, and SP1 was the key transcription factor for CXCL1, CXCL14, and CXCL5 (**Table 4**).

**Table 4:** Key regulated factor of abnormal expression CXC chemokines in PAAD (TRRUST).

	Key TF	Description	Overlapped Genes	*p*-value	FDR
1	RELA	V-rel reticuloendotheliosis viral oncogene homolog A (avian)	CXCL1, CXCL10, CXCL2, CXCL5, CXCL12, CXCL8	1.09E−07	1.71E−07
2	NFKB1	Nuclear factor of kappa light polypeptide gene enhancer in B-cells 1	CXCL1, CXCL10, CXCL2, CXCL5, CXCL12, CXCL8	1.14E−07	1.71E−07
3	SP1	Sp1 transcription factor	CXCL1, CXCL14, CXCL5	0.00683	0.00683

CXCL, C-X-C chemokine ligand; PAAD, pancreatic adenocarcinoma; TF, transcription factor; FDR, false discovery rate.

### Correlation Between CXC Chemokine and Clinicopathological Stages, and Drug–Gene Interactions of Patients With PAAD

**Figure 7:**
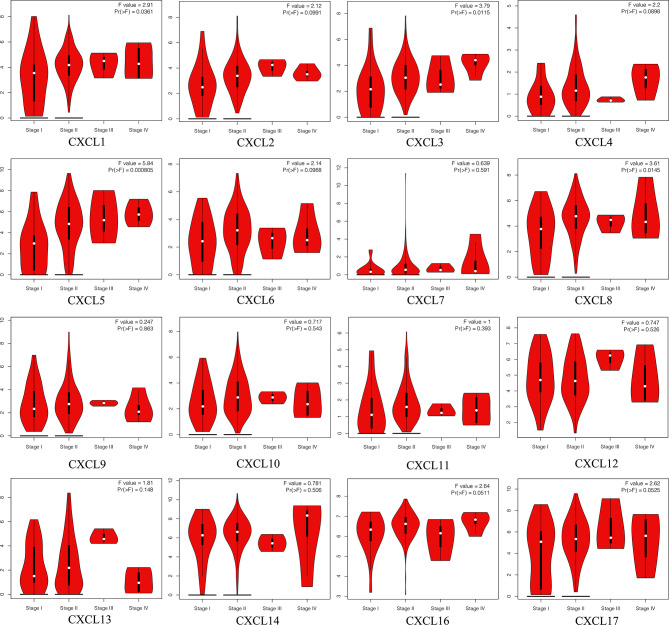
The correlation between each CXC chemokine expression and clinicopathological stages in patients with PAAD (GEPIA2).

The Pathological Stage Plot of GEPIA2 was utilized to investigate the relative transcriptional levels of CXC chemokines at different pathological stages in PAAD tissues. As shown in **Figure 7**, the expression levels of CXCL1 (*p* = 0.0361), CXCL3 (*p* = 0.0115), CXCL5 (*p* = 0.0008), and CXCL8 (*p* = 0.0145) are related to different clinicopathological stages in PAAD. In addition, of the 16 CXC chemokines, the expressions of 6 CXC chemokines show significant correlations with drug–gene interaction, namely, CXCL2, CXCL4, CXCL8, CXCL10, CXCL12, and CXCL13 (**Table 5**).

**Table 5:** The drug gene interactions or potentially available drug categories for PAAD patients with abnormal expression CXC chemokines (DGidb).

Gene	Drug	Interaction Types	PMIDs	Interaction Score
CXCL2	*Alteplase*	n/a	18199827	1.08
	*BCG Vaccine*	n/a	18217952	1.89
	*Deferoxamine*	n/a	17883261	1.51
	*Batimastat*	n/a	18477053	2.52
CXCL4	*Heparin*	n/a	20162249, 8282825	4.48
CXCL8	ABX-IL8	inhibitor		1.93
	*Foscarnet*	n/a	10630964	1.93
	Talc	n/a	17000556	1.93
CXCL10	NI-0801	inhibitor		8.74
	*Eldelumab*	n/a		13.11
	*Clove Oil*	n/a	28407719	2.18
CXCL12	*Tinzaparin*	*binder*	*18991783*	*3.55*
	*Rituximab*	*n/a*	*27173875*	*1.01*
	*Alemtuzumab*	*n/a*	*27173875*	*1.78*
	*Chlorambucil*	*n/a*	*27173875*	*1.18*
CXCL13	*Rituximab*	n/a	26384320	8.12

CXCL, C-X-C chemokine ligand; PAAD, pancreatic adenocarcinoma.

### Survival Analysis of Single CXC Chemokine in Patients With PAAD

**Figure 8:**
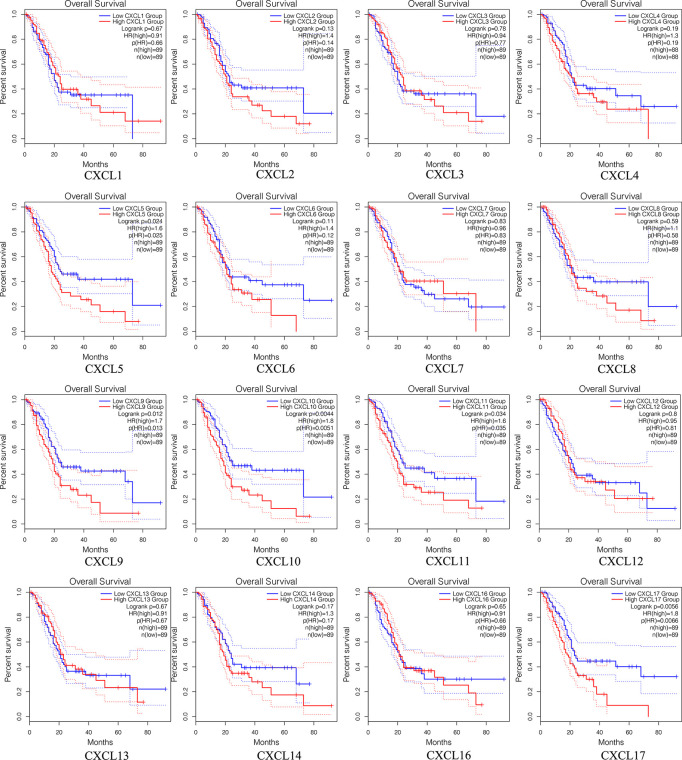
The OS of single CXC chemokine in patients with PAAD (GEPIA2).

**Figure 9:**
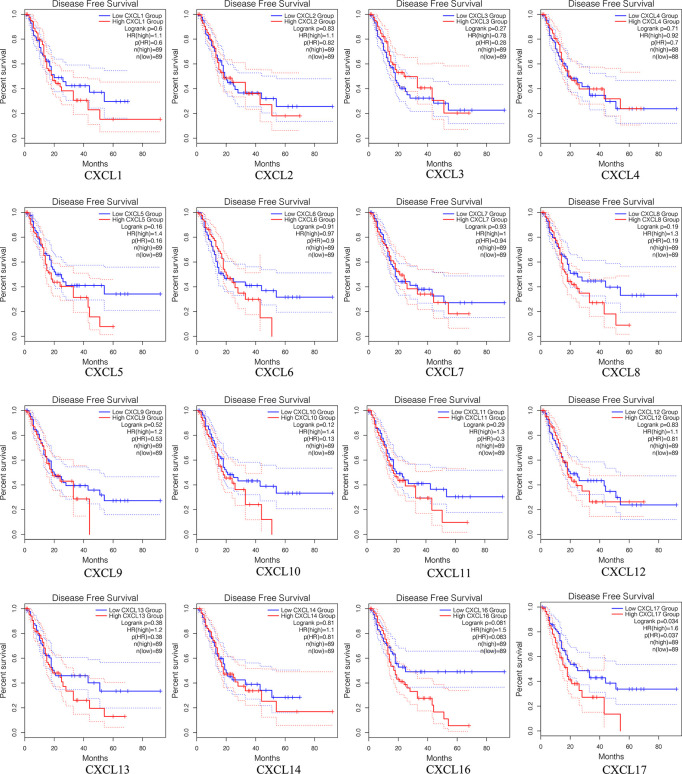
The DFS of single CXC chemokine in patients with PAAD (GEPIA2).

The prognostic value among each CXC chemokine was evaluated with GEPIA2. The expressions of CXCL5 (HR = 1.6; *p* = 0.024), CXCL9 (HR = 1.7; *p* = 0.012), CXCL10 (HR = 1.8; *p* = 0.0044), CXCL11 (HR = 1.6; *p* = 0.034), and CXCL17 (HR = 1.8; *p* = 0.0056) are connected with unfavorable OS of patients with PAAD (**Figure 8**). However, only high transcriptional levels of CXCL17 (HR = 1.6; *p* = 0,034) are an unfavorable prognostic factor of DFS in PAAD patients (**Figure 9**). The results indicated that PAAD patients with high transcriptional levels of CXCL17 had worse DFS.

### Survival Analysis of Single CpG of DNA Methylation of CXC Chemokines in Patients With PAAD

**Figure 10:**
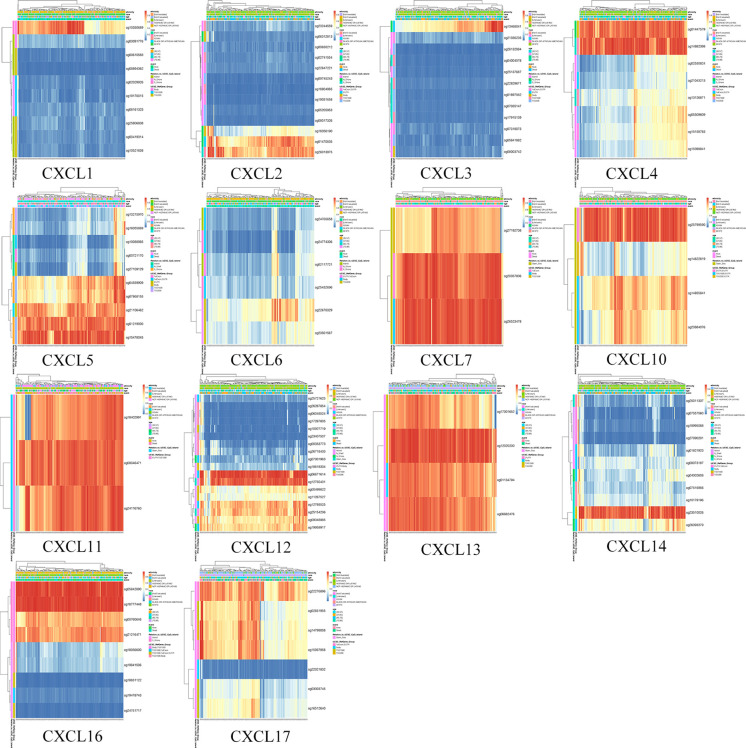
The heatmap of DNA methylation level of single CXC chemokine (MethSurv). High expression (red); low expression (blue).

The DNA methylation of 16 CXC chemokines was input for prognostic analysis in MethSurv. The heatmaps of DNA methylation of each CXC chemokine were explored and displayed in **Figure 10**. Among them, cg10350689 of CXCL1, cg26013975 of CXCL2, cg13468041 of CXCL3, cg14882398 of CXCL4, cg15478045 of CXCL5, cg22670329 of CXCL6, cg26523478 of CXCL7, cg25799590 of CXCL10, cg08046471 of CXCL11, cg06671614 of CXCL12, cg12020230 of CXCL13, cg23510026 of CXCL14, cg18777448 of CXCL16, and cg22276896 of CXCL17 showed the highest DNA methylation level. In addition, we found that 3 CpGs of CXCL1, 2 CpGs of CXCL2, 2 CpGs of CXCL3, 3 CpGs of CXCL4, 10 CpGs of CXCL5, 1 CpG of CXCL6, 1 CpG of CXCL7, 3 CpGs of CXCL12, 3 CpGs of CXCL14, and 5 CpGs of CXCL17 were significantly associated with prognosis in patients with PAAD (**Table 6** and **Figure S7**).

**Table 6:** The significantly prognostic value of single CpG of CXC Chemokine in PAAD (MethSurv).

Gene-CpG	HR	LR test *p*-value
CXCL1 − Body−S_Shore−cg10350689	0.533	0.012
CXCL1 − TSS200−Island−cg08894362	1.906	0.002
CXCL2 − TSS1500−Island−cg02741554	1.684	0.032
CXCL2 − 3’UTR−N_Shore−cg01470535	0.509	0.00096
CXCL3 − TSS200−Island−cg26132084	1.941	0.0017
CXCL3 − 1stExon;5’UTR−Island−cg21336235	1.706	0.02
PF4 − Body−N_Shore−cg01447579	1.761	0.011
PF4 − TSS200−Island−cg05509609	0.598	0.037
PF4 − 1stExon;5’UTR−Island−cg21043213	1.679	0.028
CXCL5 − 1stExon−Island−cg00721170	0.597	0.011
CXCL5 − 1stExon;5’UTR−Island−cg10088985	0.547	0.014
CXCL5 − Body−Island−cg27109129	0.593	0.019
CXCL5 − TSS1500−S_Shore−cg01219000	0.61	0.029
CXCL5 − TSS1500−S_Shore−cg04559909	0.403	0.00028
CXCL5 − TSS1500−S_Shore−cg07868155	0.425	0.00049
CXCL5 − TSS1500−S_Shore−cg15478045	1.591	0.024
CXCL5 − TSS200−S_Shore−cg13215970	0.521	0.0032
CXCL5 − TSS200−S_Shore−cg16055869	0.462	0.0013
CXCL5 − 3’UTR−N_Shelf−cg21106462	0.496	9.0e−04
CXCL6 − Body−S_Shore−cg25432696	0.613	0.043
PPBP − 1stExon−Open_Sea−cg20357806	0.648	0.048
CXCL12 − Body−N_Shelf−cg12793525	0.639	0.032
CXCL12 − TSS1500−Island−cg09348985	2.27	0.0011
CXCL12 − TSS1500−Island−cg11267527	1.695	0.031
CXCL14 − 5’UTR;1stExon−Island−cg18995088	0.644	0.046
CXCL14 − TSS1500−S_Shore−cg23510026	1.546	0.033
CXCL14 − TSS200−S_Shore−cg07516956	0.656	0.038
CXCL17 − 1stExon;5’UTR−Open_Sea−cg15937958	0.417	3.7e−05
CXCL17 − 1stExon;5’UTR−Open_Sea−cg22276896	0.388	4.5e−05
CXCL17 − TSS200−Open_Sea−cg14799008	0.446	0.0018
CXCL17 − TSS1500−Open_Sea−cg02831955	0.525	0.0018
CXCL17 − TSS1500−Open_Sea−cg03003745	1.618	0.041

CXCL, C-X-C chemokine ligand; PAAD, pancreatic adenocarcinoma; HR, hazard ratio; LR, test likelihood-ratio test.

### Survival Analysis of CXC Chemokine Signature in Patients With PAAD

**Figure 11:**
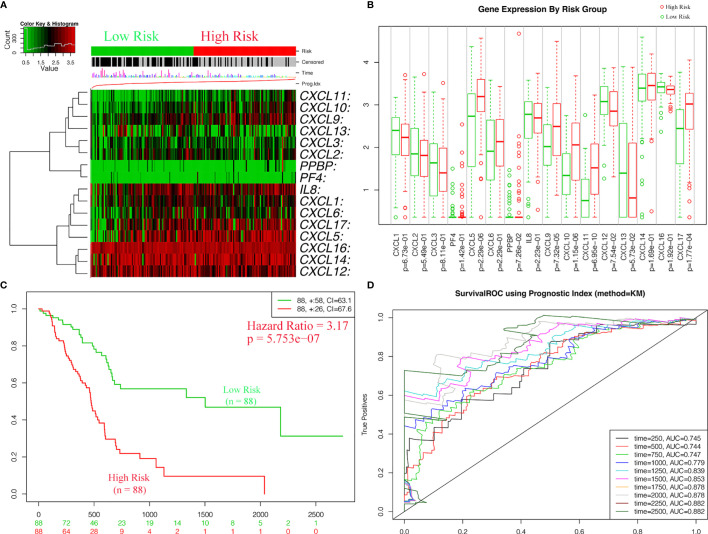
The expressions levels and prognostic values of CXC chemokine signature in the training cohort (SurvExpress). **(A)** The heatmap of CXC chemokines in PAAD patients in the high- and low-risk group. **(B)** The expression levels of CXC chemokines between high- and low-risk groups. **(C)** Kaplan–Meier curves for survival analysis of CXC chemokines between high- and low-risk groups. **(D)** The survival ROC curves for survival prediction by the CXC chemokines assessed the accuracy of the prognostic model.

For the purpose of exploring whether CXC chemokine overexpression was significantly correlated with a poor prognosis in PAAD, the expression levels of CXC chemokines were evaluated based on the TCGA dataset (**Figure 11A**). In addition, patients in the high-risk group revealed an obviously higher expression levels of CXCL5 (*p* = 2.29e−06), CXCL9 (*p* = 7.32e−05), CXCL10 (*p* = 1.15e−06), CXCL11 (*p* = 6.95e−10), and CXCL17 (*p* = 1.77e−04) (**Figure 11B**). Meanwhile, the relationship between clinicopathological characteristics of PAAD and risk score was exhibited in **Table 7**. The statistical analysis shows that the risk score-based CXC chemokine signature was related to age (*p* = 0.002), histological grade (*p* = 0.008), and survival status (*p* = 0.013). The effectiveness of prognostic prediction of the combined CXC chemokines in PAAD patients is explored by Kaplan–Meier survival analysis based on SurvExpress. As shown in **Figure 11C**, compared with the low-risk group, patients in the high-risk group suffered from poor prognosis (*p* = 5.753e−07, HR = 3.17, 95% CI 2.02–4.99). The ROC curves for survival prediction by the CXC chemokines assessed the accuracy of the prognostic model (**Figure 11D**). Similarly, these results were also confirmed in the validation cohort (ICGC—Pancreatic ductal adenocarcinoma (42), 189 patients, June 2016). As expected, the patients in the low-risk group had a better OS compared with the high-risk group (**Figure S8**).

**Table 7:** Clinicopathological features of the PAAD patients in TCGA cohort and the relationship between clinicopathological features and CXC chemokine signature (TCGA and SurvExpress).

Characteristics	Variable	*n* (%)	Risk score based on CXC chemokine signature		*χ* ^2^	*p*
			High-Risk	Low-Risk		
Age	<=65	93 (52.8%)	36	57	10.055	0.002
	>65	83 (47.2%)	52	31		
Gender	Male	97 (55.1%)	44	53	1.860	0.225
	Female	79 (44.9%)	44	35		
Histological grade	G1	30 (17.1%)	11	19	12.028^a^	0.008
	G2	95 (54%)	44	51		
	G3	47 (26.7%)	33	14		
	G4	2 (1.1%)	1	1		
	Not available	2 (1.1%)	2	0		
Stage	I	21 (11.9%)	7	14	4.196^a^	0.407
	II	145(82.4%)	79	66		
	III	3 (1.7%)	2	1		
	IV	4 (2.3%)	2	2		
	Not available	3 (1.7%)	1	2		
T classification	T1	7 (4%)	3	4	2.842^a^	0.637
	T2	24 (13.6%)	10	14		
	T3	140 (79.6%)	76	64		
	T4	3 (1.7%)	2	1		
	Not available	2 (1.1%)	0	2		
M classification	M0	79 (44.9%)	45	34	2.082^a^	0.328
	M1	4 (2.3%)	2	2		
	Not available	93 (52.8%)	43	50		
N classification	N0	49 (27.9%)	21	28	4.532^a^	0.115
	N1	122 (69.3%)	69	53		
	Not available	5 (2.8%)	1	4		
Survival status	Dead	84 (47.7%)	29	55	6.181	0.013
	Alive	92 (52.3%)	62	30		

a denotes that Fisher’s exact test was applied when there were at least one expected count less than 5.

CXCL, C-X-C chemokine ligand; PAAD, pancreatic adenocarcinoma; TCGA, The Cancer Genome Atlas.

### Survival Analysis of the DNA Methylation of CXC Chemokine Signature

**Figure 12:**
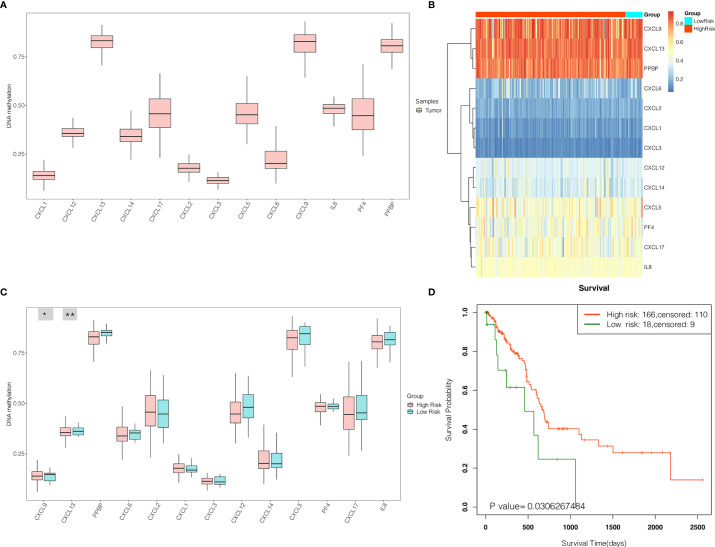
The expression levels and prognostic values of the DNA methylation of CXC chemokine signature in patients with PAAD (SurvivalMeth). **(A)** The DNA methylation level of single CXC chemokine in PAAD patients. **(B)** The heatmap of DNA methylation of CXC chemokines in PAAD patients in high- and low-risk groups. **(C)** The expression levels of DNA methylation of CXC chemokines between high- and low-risk groups. *p < 0.05; **p < 0.01. **(D)** Kaplan–Meier curves for survival analysis of DNA methylation of CXC chemokines between high- and low-risk groups.

In order to further validate the prognostic power, CXC chemokines were input for survival analysis of the DNA methylation of CXC chemokine signature in SurvivalMeth. The DNA methylation level of CXC chemokines was explored and displayed in **Figures 12A, B**. Furthermore, our results revealed that the DNA methylation level of CXCL13 was the highest, while CXCL3 was the lowest. As shown in **Figure 12C**, the differential DNA methylation level was found in CXCL9 and CXCL13 between the high- and the low-risk group. Moreover, the survival analysis demonstrated that PAAD patients in the high-risk group had longer survival times (*p* < 0.05, **Figure 12D**).

### Validation of the Expression Levels and Prognostic Power of CXC Chemokines Using the GEO Database

**Figure 13:**
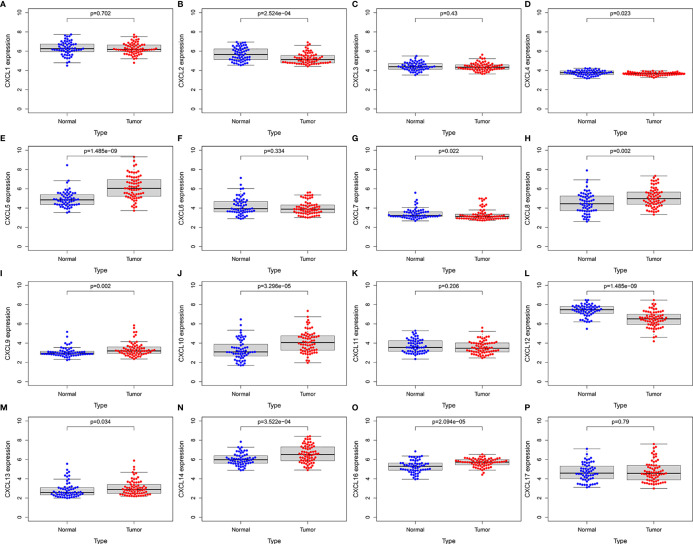
External validation of the expression levels of CXC chemokines in independent GSE62452 cohort (GEO database). **(A)** CXCL1 **(B)** CXCL2 **(C)** CXCL3 **(D)** CXCL4 **(E)** CXCL5 **(F)** CXCL6 **(G)** CXCL7 **(H)** CXCL8 **(I)** CXCL9 **(J)** CXCL10 **(K)** CXCL11 **(L) **CXCL12 **(M)** CXCL13 **(N)** CXCL14 **(O)** CXCL16 **(P)** CXCL17.

To further evaluate the expression levels and prognostic power of CXC chemokines in other datasets, the external gene expression profile GSE62452 with 69 pancreatic cancer samples and 61 normal pancreatic tissue was downloaded with its associated follow-up information from the GEO database for validation of the expression levels and prognostic power of CXC chemokines. Similar to the results in TCGA datasets, the expression levels of CXCL5/8/9/10/13/14/16 were significantly elevated in pancreatic cancer tissues, while the expression levels of CXCL2/4/7/12 were significantly reduced in tumor tissues, and there was no noticeable discrepancy in the expression of CXCL1/3/6/11/17 between the pancreatic cancer tissues and the normal pancreatic tissues (**Figure 13**). Furthermore, the prognostic power of CXC chemokines in the GEO database was also verified. The K–M curves demonstrated that a low expression level of CXCL5 was associated with a better prognosis in pancreatic cancer (**Figure S9**). One likely reason of discordant findings was attributed to insufficient sample size.

## Discussion

Pancreatic cancer remains one of the top five principal causes of cancer-related death and one of the most aggressive tumors despite the remarkable improvements in multimodality therapeutic strategies (1, 43). Therefore, further research on the potential therapeutic targets for PAAD patients is in need. The CXC chemokines, a subfamily of functional chemotactic peptides, were initially defined as inflammatory mediators that contribute to the differentiation, maturation, and attractants of leukocytes, in particular, during inflammation and infection (44). Increasing studies have identified that CXC chemokines, expressed by several tumor cell types, contribute to tumor-related processes, including tumorigenesis, TME, tumor recurrence, local invasion, and metastasis (45, 46). In addition, members of the CXC chemokines also contain the ELR^+^ (Glu‐Leu‐Arg) amino acid motif and play a crucial role in the regulation of tumor-related angiogenesis and in tumor proliferation and sustenance (47). We summarize the features and functions of CXC chemokines in pancreatic cancer (27, 46, 48–82) (**Table 8**). To date, the biological characteristics, functions, and clinical significance of CXC chemokines in PAAD have not been clarified.

**Table 8:** CXC chemokines and their functions in pancreatic cancer.

Chemokine;	Original name	ELR	Receptor(s)	Function in pancreatic cancer	Reference
(Cytoband)					
CXCL1	**GRO-*α*** growth-related oncogene *α*	Position	CXCR2	Chemoresistance, proliferation, angiogenesis, carcinogenesis, inflammation, immune suppression	(50–57)
(4q13.3)					
CXCL2	**GRO-*β*** growth-related oncogene *β*	Position	CXCR2	Inflammation, proliferation, migration	(51–58)
(4q13.3)					
CXCL3	**GRO-*γ*** growth-related oncogene *γ*	Position	CXCR2	Tumor progression, Inflammation	(59)
(4q13.3)					
CXCL4	**PF-4** platelet factor 4	Negative	CXCR3	Anti-angiogenesis, growth inhibition	(46, 47, 50–60)
(4q13.3)					
CXCL5	**ENA-78** Epithelial cell-derived neutrophil activating factor 78	Position	CXCR2	Chemoresistance, tumorigenesis, invasion, angiogenesis, proliferation, migration, immune cell infiltration	(61–64)
(4q13.3)					
CXCL6	**GCP-2** Granulocyte chemoattractant protein 2	Position	CXCR1/CXCR2	Angiogenesis, inflammation, metastasis	(48, 56–60)
(4q13.3)					
CXCL7	**NAP-2** neutrophil-activating protein 2	Position	CXCR2	Angiogenesis, immune cell infiltration	(65)
(4q13.3)					
CXCL8	**IL-8** interleukin 8	Position	CXCR1/CXCR2	Chemoresistance, angiogenesis, immune cell infiltration, proliferation, migration,	(66–69)
(4q13.3)					
CXCL9	**MIG** monokine-induced by interferon *γ*	Negative	CXCR3	Immunosensitivity, apoptosis, anti-angiogenesis, growth inhibition	(69, 70)
(4q21.1)					
CXCL10	**IP-10***γ* interferon inducible protein 10	Negative	CXCR3	Immune cell infiltration, immunosuppression, tumor progression, chemoresistance, anti-migration	(69, 71–74)
(4q21.1)					
CXCL11	**I-TAC** Interferon inducible T cell a chemoattractant	Negative	CXCR3	Angiogenesis, proliferation	(75)
(4q21.1)					
CXCL12	**SDF-1** Stromal cell-derived factor 1	Negative	CXCR4	Proliferation, invasion, migration, chemoresistance, angiogenesis	(76)
(10q11.21)					
CXCL13	**BCA-1** B cell-activating chemokine 1	Negative	CXCR5	Tumorigenesis, proliferation	(77)
(4q21.1)					
CXCL14	**BRAK** breast and kidney chemokine	Negative	Unknown	Angiogenesis, growth, migration, invasion, immune cell infiltration	(78, 79)
(5q31.1)					
CXCL16	**SR-PSOX** scavenger receptor that binds phosphatidylserine and oxidized lipoprotein	Negative	CXCR6	Angiogenesis, growth, migration, invasion	(80, 81)
(17p13.2)					
CXCL17	**VCC-1** VEGF- correlated chemokine-1	Negative	Unknown	Immune surveillance	(49)
(19q13.2)	**DMC** dendritic cell and monocyte-like protein				

CXCL, C-X-C chemokine ligand; CXCR, C-X-C chemokine receptor type; ELR, Glu‐Leu‐Arg.

Tumor cells interact closely with CXC chemokines and TME. *Via* complex mechanisms, these communications were involved in tumor growth, immune escape, and therapeutic efficacy (83). Intercellular communication between CXC chemokines and TME affects the patterns of proliferation and apoptosis in various tumor cell types, thus facilitating specific biological mechanism for tumor invasion and metastasis (14). In the TME, CXC chemokines regulate tumor angiogenesis, modulate inflammation and immunity, and participate in the formation of non-vascular tumor stroma. The manipulation of CXC chemokine–chemokine receptor signaling can reshape the immunological phenotypes within the TME in order to increase the therapeutic efficacy of cancer immunotherapy.

Tumor angiogenesis is an important pathological process in the initiation and progression of PAAD (9, 83). The new blood vessel not only delivers nutrients and oxygen but also removes waste products. In the context of tumor angiogenesis, the CXC chemokines can promote or inhibit tumor angiogenesis under certain conditions (84). The CXC chemokines containing the ELR^+^ are the promoters of tumor angiogenesis (47, 50, 85), which can interact with their receptors on blood vessel endothelial cells and attract inflammatory cells that release angiogenic factors such as vascular endothelial growth factor (VEGF), platelet-derived growth factor (PDGF), and fibroblast growth factor (FGF) (84). CXCL1/2/3/5/6/7/8 (receptors are CXCR2) have been demonstrated to be a promoter in tumor angiogenesis within a number of cancer types, including pancreatic cancer, melanoma, and prostate cancer (50, 86). The results revealed that CXCR2 is a potential anti‐angiogenic target in malignant tumors. On the contrary, CXCL4, CXCL9, CXCL10, CXCL11, CXCL12, CXCL13, and CXCL14, lacking the ELR amino acid motif (ELR^−^), can inhibit tumor angiogenesis (47, 87, 88).

Many cancer types show altered CXC chemokine expression profiles, favoring the recruitment of pro-tumorigenic immune cells and preventing the accumulation of anti-tumorigenic effector cells (46, 48, 84). High levels of CXC chemokine are correlated with immune dysfunction in pancreatic cancer (49). One explanation for the immune dysfunction is that altered CXC chemokine expression profiles in TME can increase the recruitment of pro-tumorigenic immune cells such as myeloid-derived suppressor cells (MDSCs), tumor-associated neutrophils (TANs), tumor-associated macrophages (TAMs), and regulatory T cells (Treg) *via* the CXCL–CXCR axes (50, 89). For instance, overexpression of CXCL5 in pancreatic cancer cells promoted TAN recruitment and correlated with worse prognosis through the CXCL5–CXCR2 axes (61–64). Immune dysfunction plays an essential role in tumor progression and tumor invasion. The expression of CXC chemokines can influence the progression of malignant tumors by forming the infiltrating immune cell population. Ali et al. (90). presented that the cytotoxic natural killer (NK) cells were assembled in lymph nodes, induced by the expression of CXCL8, and played an essential role in the progression of melanoma to peripheral lymph nodes metastasis, infiltration, and invasion. In addition, high CXCL10 expression in the TME can prolong NK cell-dependent survival. CXCL9, CXCL10, and CXCL12 are important chemotactic factors in the TME, which are able to recruit T cells and increase inflammation in tumors such as melanoma and lung adenocarcinoma (29, 91). Importantly, it was recently shown that CXCL–CXCR axes within the TME display high plasticity.

In this study, the relationship between CXC chemokines and clinical significance in PAAD was explored using several bioinformatics analysis tools for a better understanding of the initiation and progression in PAAD. The findings showed that most CXC chemokines were upregulated in PAAD, which were correlated with clinical outcomes. These findings were in accordance with the results of expression levels reported by Iacobuzio-Donahue et al. (30), who found a significant increase of CXCL2 (*p* = 0.047), CXCL3 (*p* = 0.013), CXCL6 (*p* = 0.012), CXCL8 (*p* = 0.01), CXCL9 (*p* = 0.026), CXCL14 (*p* = 1.49E−5), and CXCL16 (*p* = 0.003) in PAAD. Buchholz et al. (31) also presented that the expression levels of CXCL2 (*p* = 0.001) and CXCL9 (*p* = 6.21E−4) in pancreatic ductal adenocarcinoma significantly increased. The datasets of Badea et al. (32) also suggested that CXCL2 (*p* = 0.001), CXCL3 (*p* = 3.8E−7), CXCL5 (*p* = 3.37E−13), CXCL6 (*p* = 1.85E−6), CXCL8 (*p* = 9.92E−12), CXCL9 (*p* = 1.27E−5), CXCL13 (*p* = 0.01), CXCL14 (*p* = 1.51E−6), and CXCL16 (*p* = 6.64E−12) significantly rose in pancreatic ductal adenocarcinoma compared with normal samples. Similarly, moreover, the research of Pei et al. (33), Logsdon et al. (34), Ishikawa et al. (35), Segara et al. (36), and Grützmann et al. (37) also supported the results of this study (**Table 1**). Through drug target network analysis, it was found that several drugs, such as Alteplase, Heparin, ABX-IL8, NI-0801, and Rituximab, were associated with CXCL2, CXCL4, CXCL8, CXCL10, and CXCL13, which may be potential therapeutic targets and drugs for PAAD. However, further verification using biological experiments is needed to clarify the results of bioinformatics analysis. The survival analysis demonstrated that both CXC chemokines and DNA methylation of CXC chemokines are potential prognostic markers in patients with PAAD. The study also demonstrated that most CXC chemokines were actively involved in the tumor inflammatory responses and the infiltration of immune cells during PAAD initiation and progression, indicating that CXC chemokines not only act as prognostic indicators but also reflect immune status.

There are several limitations of the study. Firstly, the study simply relied on bioinformatics analysis, and there were no biological experiments to verify the results. Secondly, molecular mechanism and functions of CXC chemokines were under-researched. Thirdly, there were no bioinformatics analysis platforms for exploring the efficiency of PAAD-related drug targets.

## Conclusions

In conclusion, our findings reveal the novel insights into CXC chemokine expression and their biological functions in pancreatic cancers, which provide a breakthrough for the exploration of prognosis biomarkers and immunotherapeutic targets for patients with pancreatic cancer. Further work is required to systemically understand the biological functions and clinical significance of CXC chemokines in PAAD.

## Data Availability Statement

The original contributions presented in the study are included in the article/**Supplementary Material**. Further inquiries can be directed to the corresponding authors.

## Author Contributions

All authors contributed to the article and approved the submitted version. JH and ZC downloaded and analyzed the data. DW and KR designed the study. JH, ZC, and CD drafted the manuscript. DW and CD reviewed and revised the manuscript. SL and CD edited the figures and tables of the article. All authors made substantial contributions to the conception and design, acquisition of data, or analysis and interpretation of data; took part in drafting the article or revising it critically for important intellectual content; agreed to submit to the current journal; gave final approval of the version to be published; and agree to be accountable for all aspects of the work.

## Conflict of Interest

The authors declare that the research was conducted in the absence of any commercial or financial relationships that could be construed as a potential conflict of interest.

## Publisher’s Note

All claims expressed in this article are solely those of the authors and do not necessarily represent those of their affiliated organizations, or those of the publisher, the editors and the reviewers. Any product that may be evaluated in this article, or claim that may be made by its manufacturer, is not guaranteed or endorsed by the publisher.

## Supplementary Material

Supplementary Figure 1 The protein expression of CXCL4/5/7/8/11/12/13/14/16 in normal pancreatic tissues and PAAD tissues (the Human Protein Atlas).

Supplementary Figure 2 The correlation among each CXC chemokine (SurvivalMeth).

Supplementary Figure 3 Correlations between the expression of CXC chemokines and BRCA1 mutated genes (cBioportal).

Supplementary Figure 4 Correlations between the expression of CXC chemokines and BRCA2 mutated genes (cBioportal).

Supplementary Figure 5 The pathway and process enrichment analysis of CXC chemokines (Metascape). **(A)** Bar graph of enriched terms across input CXC chemokines, colored by p-values. **(B)** The top-level Gene Ontology biological processes. **(C)** Network of enriched terms, colored by cluster ID, **(D)** Network of enriched terms, colored by p-value.

Supplementary Figure 6 The comparison of tumor infiltration levels among PAAD with different somatic copy number alterations for CXC chemokines (TIMER).

Supplementary Figure 7 Kaplan–Meier curves for visualizing single CpG of CXC Chemokine together with their prognostic value in PAAD (MethSurv).

Supplementary Figure 8 The expressions levels and prognostic values of CXC chemokines signature in validation cohort (SurvExpress). **(A)** The heatmap of CXC chemokines in PAAD patients in high- and low-risk group. **(B)** The expression levels of CXC chemokines between high- and low-risk groups. **(C)** Kaplan–Meier curves for survival analysis of CXC chemokines between high- and low-risk groups. **(D)** The survivalROC curves for survival prediction by the CXC chemokines assessed the accuracy of prognostic model.

Supplementary Figure 9 External validation of the prognostic value of single CXC chemokine in independent GSE62452 cohort (GEO database).

## Abbreviations

PAAD, pancreatic adenocarcinoma; OS, overall survival; DFS, disease-free survival; TME, tumor microenvironment; GPCRs, G-protein-coupled receptors; ECM, extracellular matrix; GO, Gene Ontology; KEGG, Kyoto Encyclopedia of Genes and Genomes; PPI, protein–protein interaction; FC, fold change; FDR, false discovery rate; TCGA, the Cancer Genome Atlas database; PDAC, pancreatic ductal adenocarcinoma; BP biological processes; MF, molecular function; CC, cellular component; ELR, Glu‐Leu‐Arg; VEGF, vascular endothelial growth factor; PDGF, platelet-derived growth factor; FGF, fibroblast growth factor; NK, natural killer.
