# Supplementary figures and images for: Prognostic Biomarkers and Immunotherapeutic Targets Among CXC Chemokines in Pancreatic Adenocarcinoma
Source: Front Oncol. 2021 Aug 23;11:711402. doi: 10.3389/fonc.2021.711402 (PMC8419473; doi:10.3389/fonc.2021.711402)

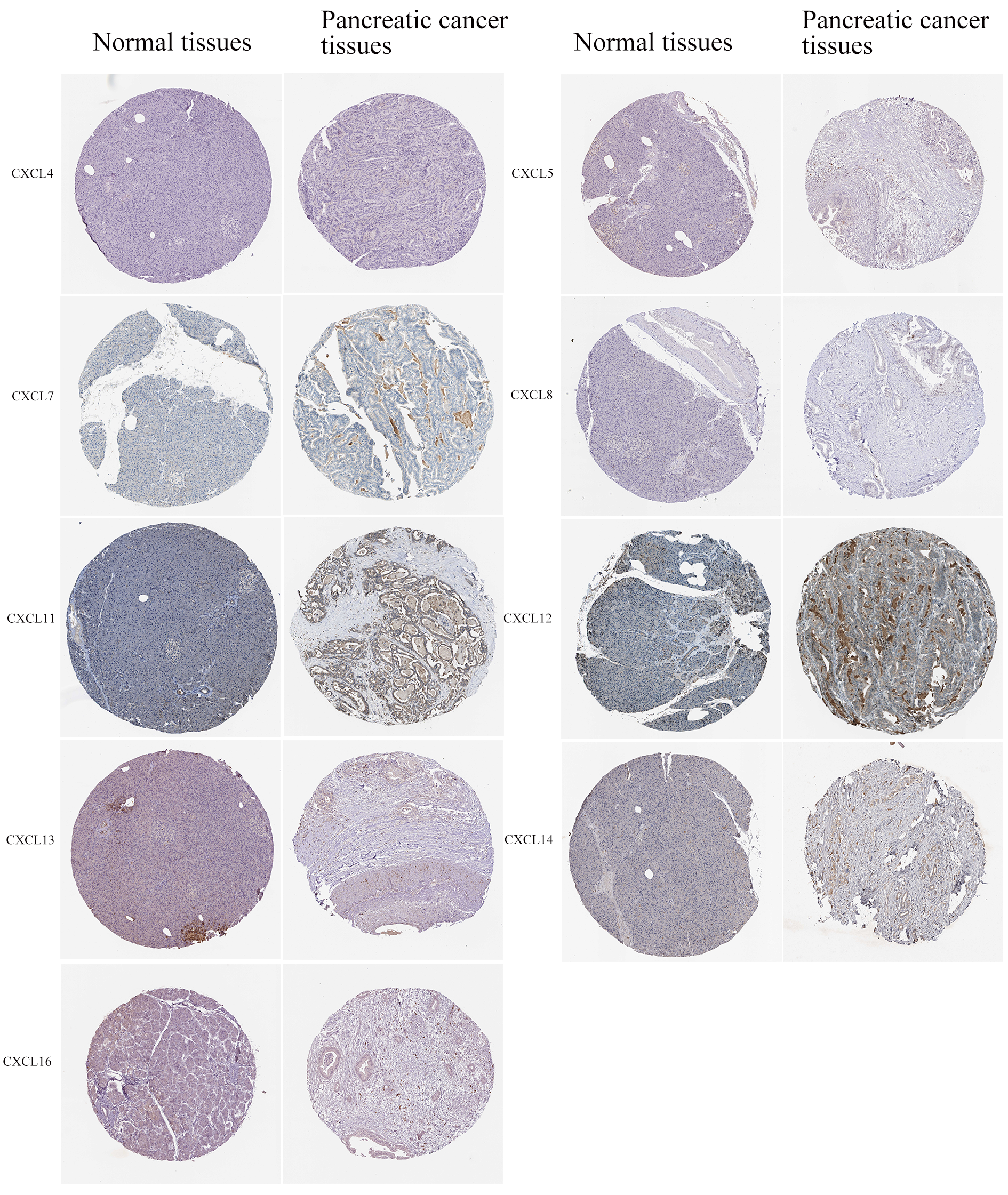

Supplement: Supplementary Figure 1 — The protein expression of CXCL4/5/7/8/11/12/13/14/16 in normal pancreatic tissues and PAAD tissues (the Human Protein Atlas). [file Image_1.tif]

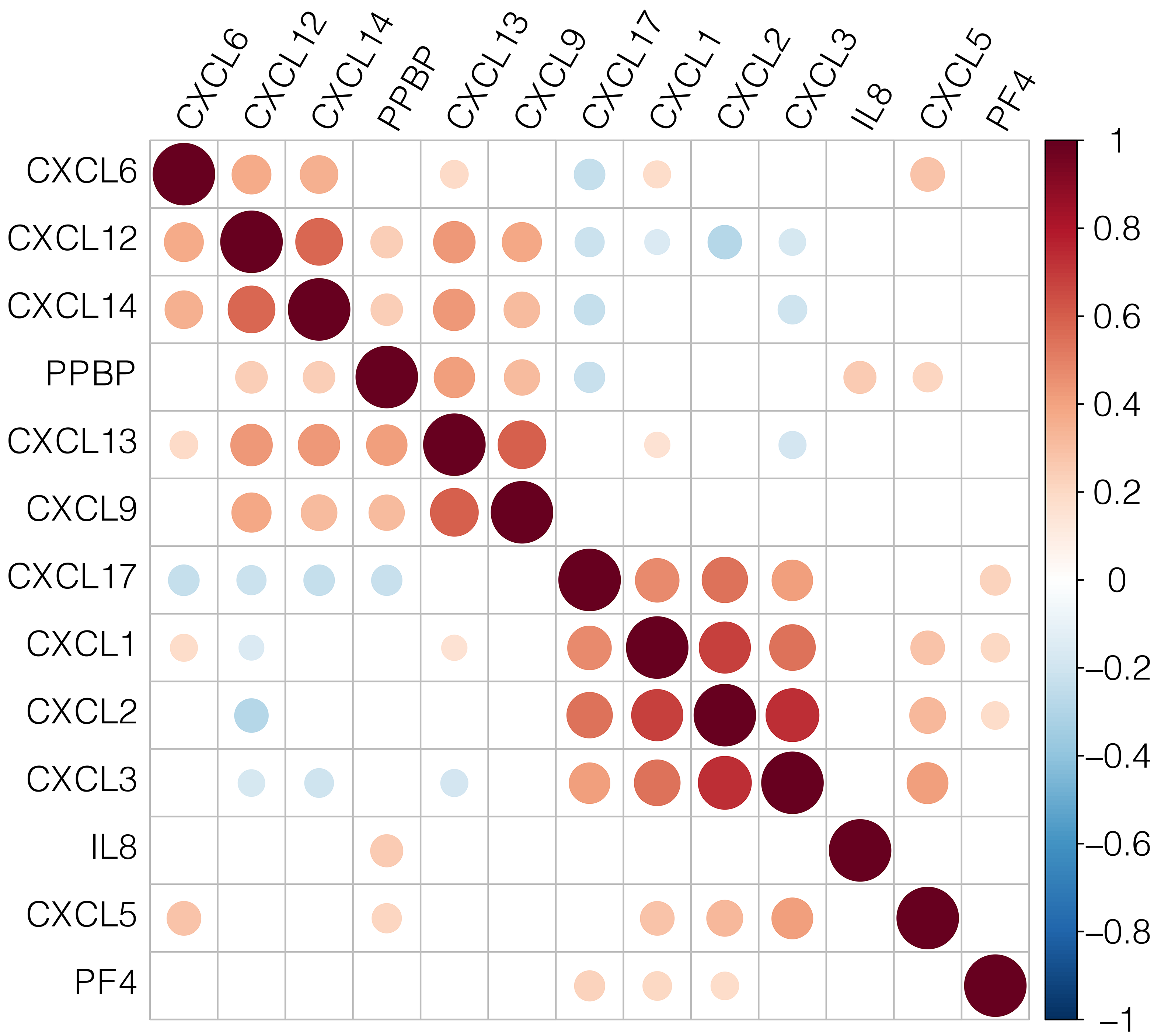

Supplement: Supplementary Figure 2 — The correlation among each CXC chemokine (SurvivalMeth). [file Image_2.tif]

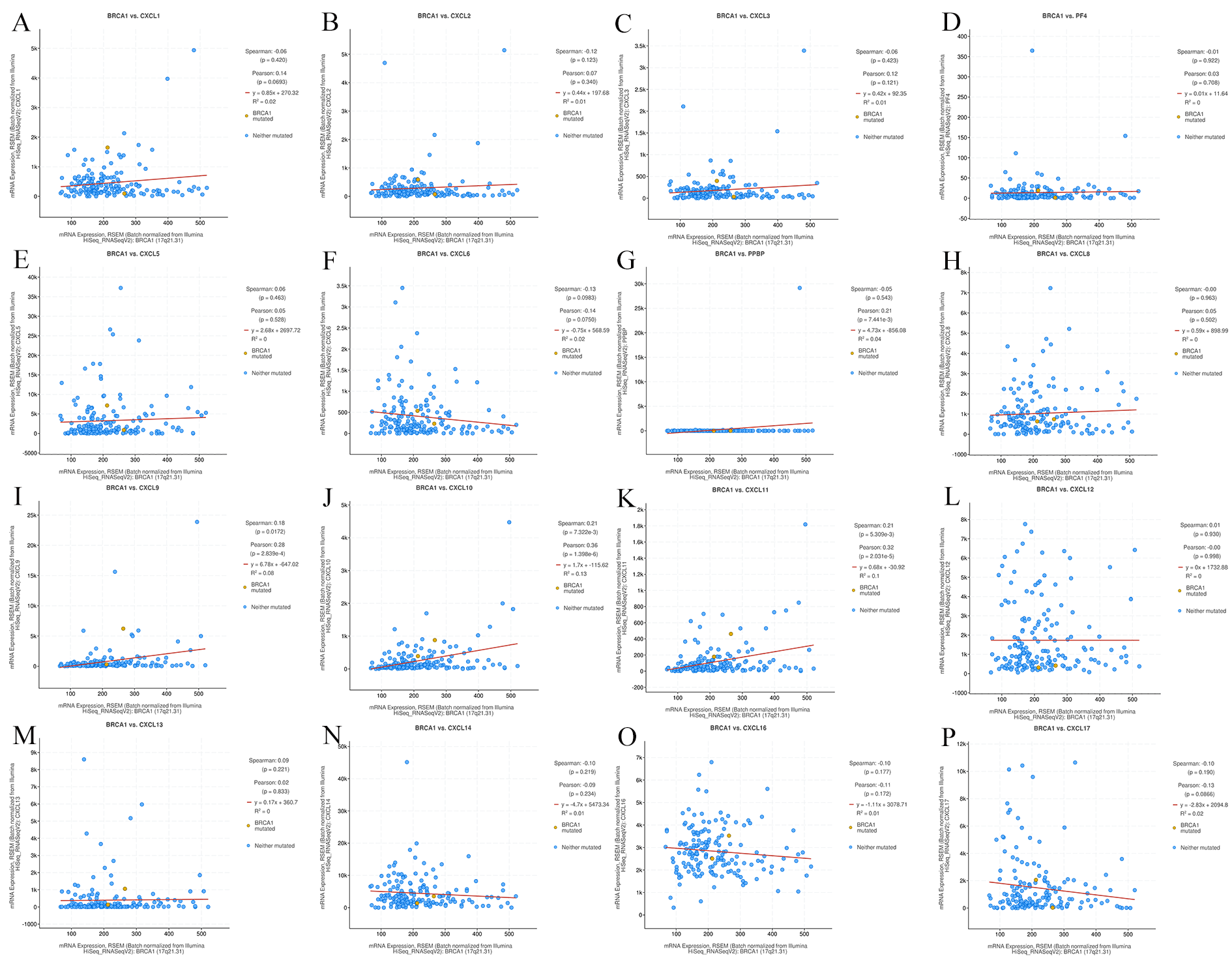

Supplement: Supplementary Figure 3 — Correlations between the expression of CXC chemokines and BRCA1 mutated genes (cBioportal). [file Image_3.tif]

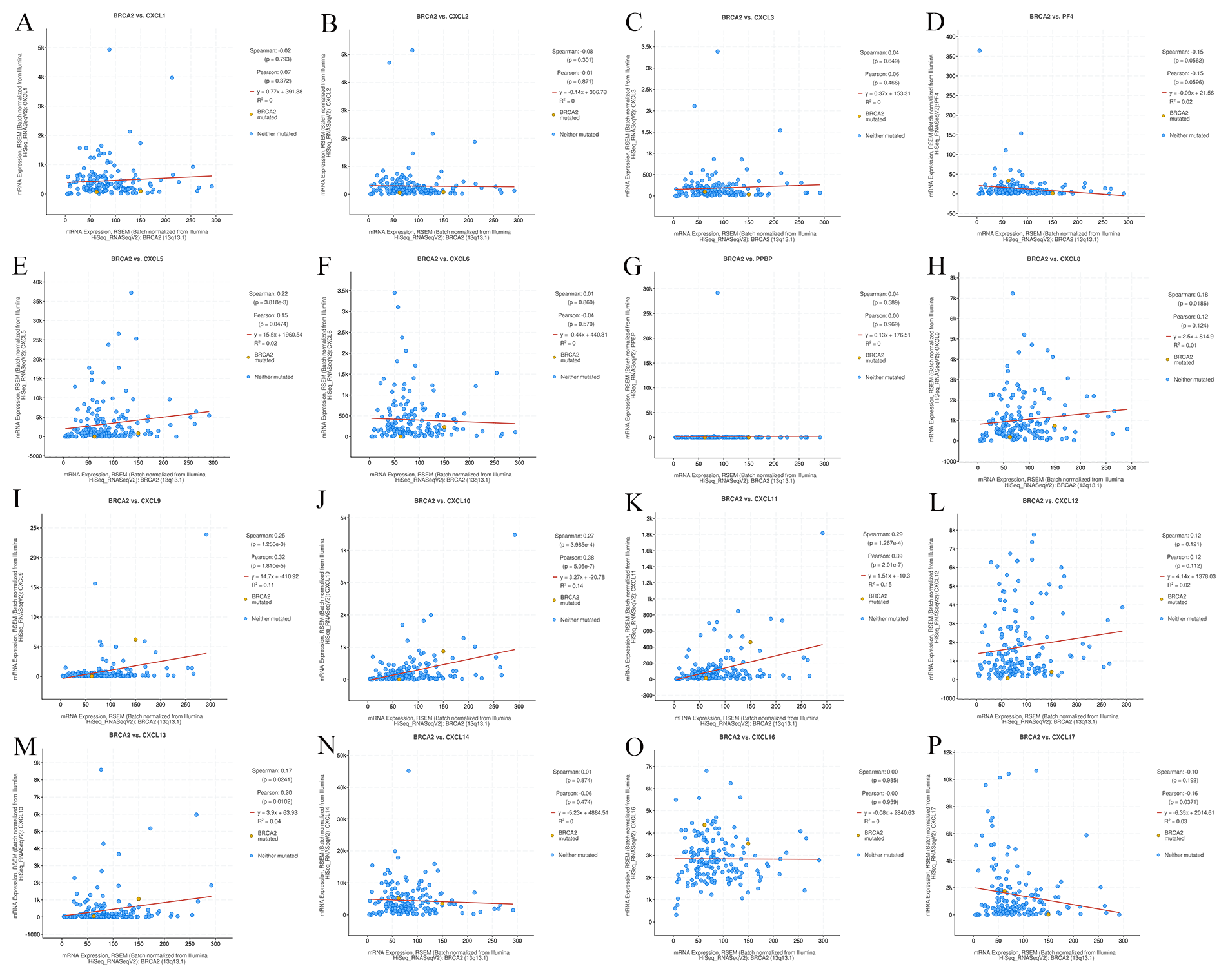

Supplement: Supplementary Figure 4 — Correlations between the expression of CXC chemokines and BRCA2 mutated genes (cBioportal). [file Image_4.tif]

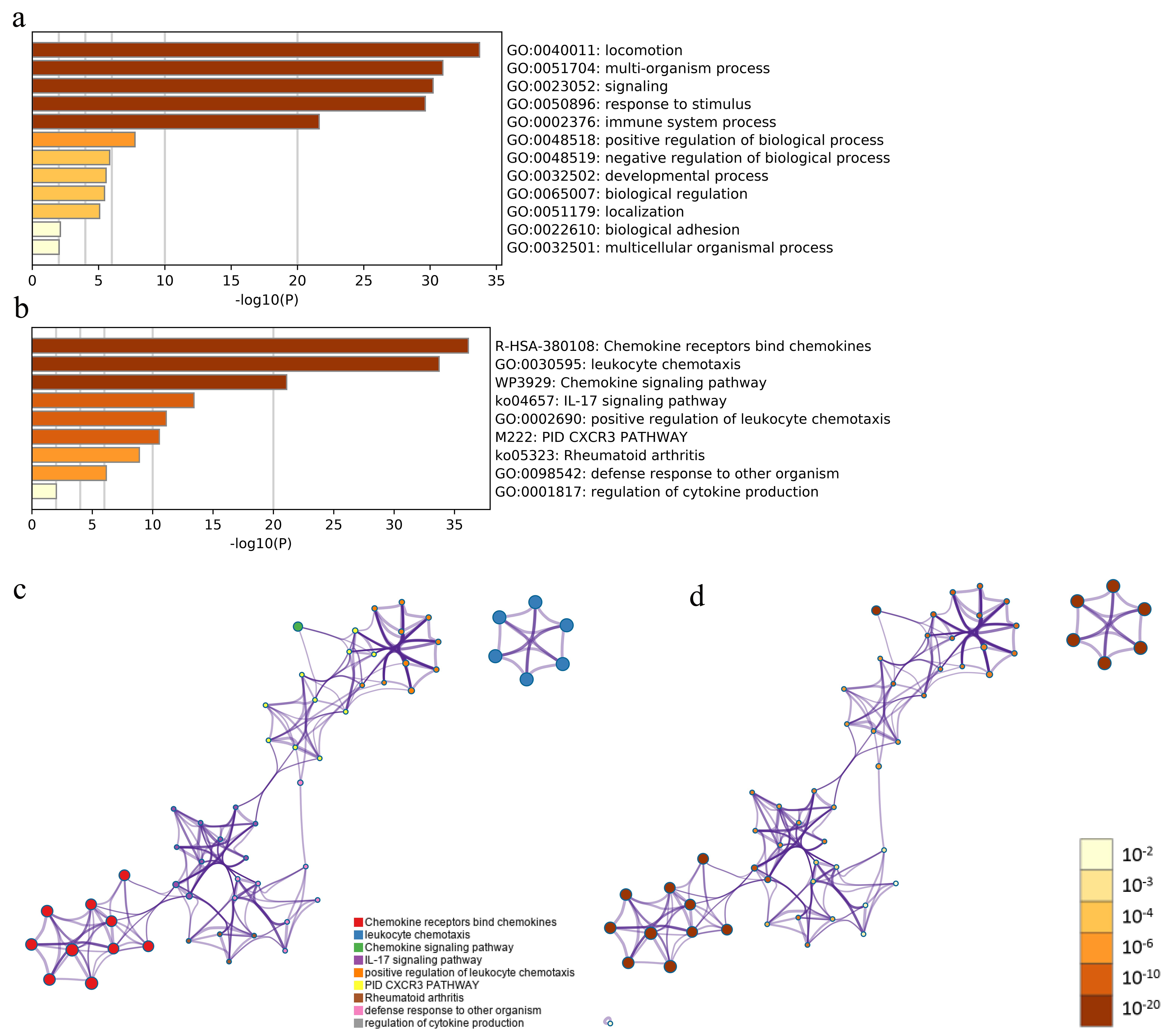

Supplement: Supplementary Figure 5 — The pathway and process enrichment analysis of CXC chemokines (Metascape). (A) Bar graph of enriched terms across input CXC chemokines, colored by p-values. (B) The top-level Gene Ontology biological processes. (C) Network of enriched terms, colored by cluster ID, (D) Network of enriched terms, colored by p-value. [file Image_5.tif]

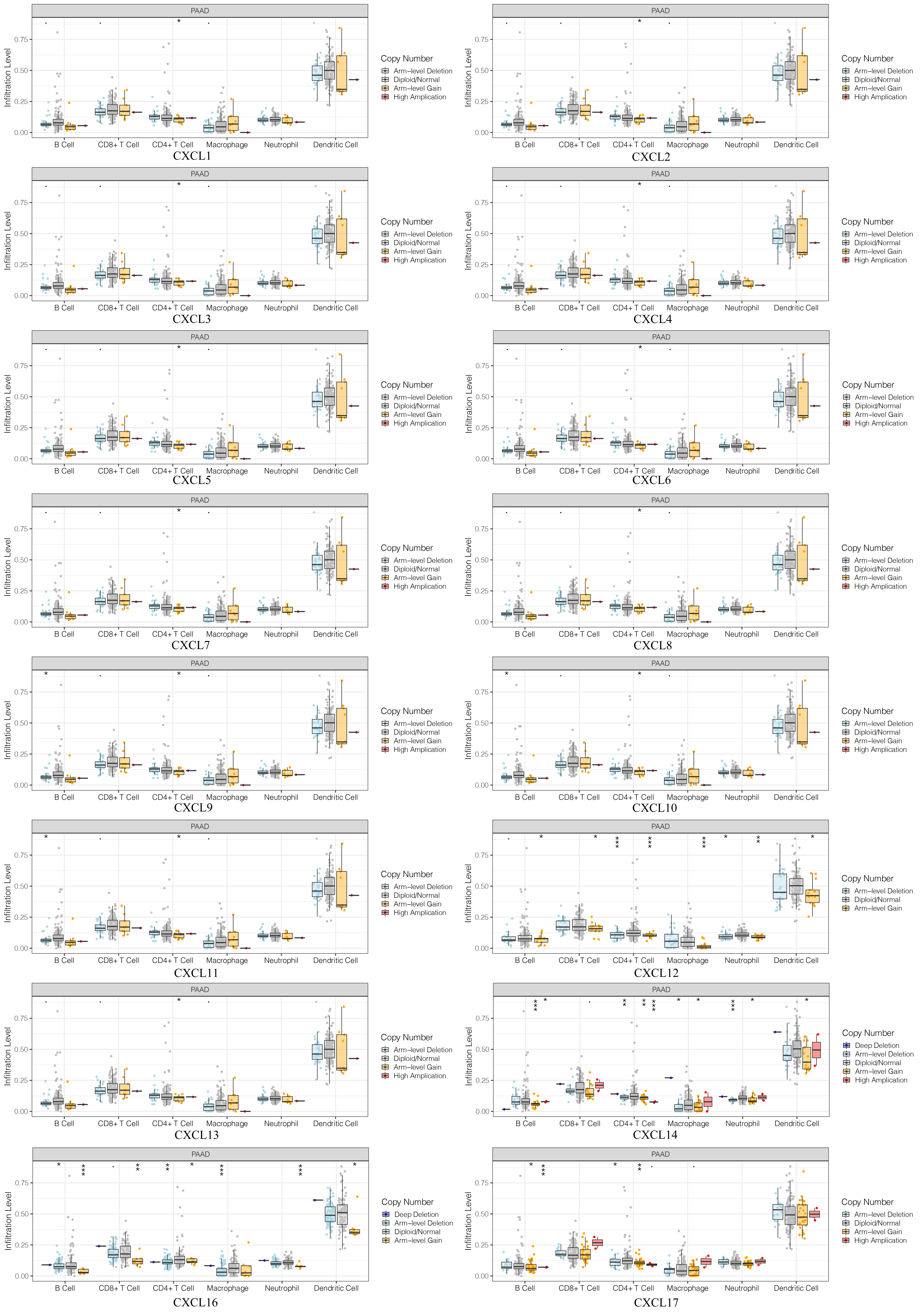

Supplement: Supplementary Figure 6 — The comparison of tumor infiltration levels among PAAD with different somatic copy number alterations for CXC chemokines (TIMER). [file Image_6.tif]

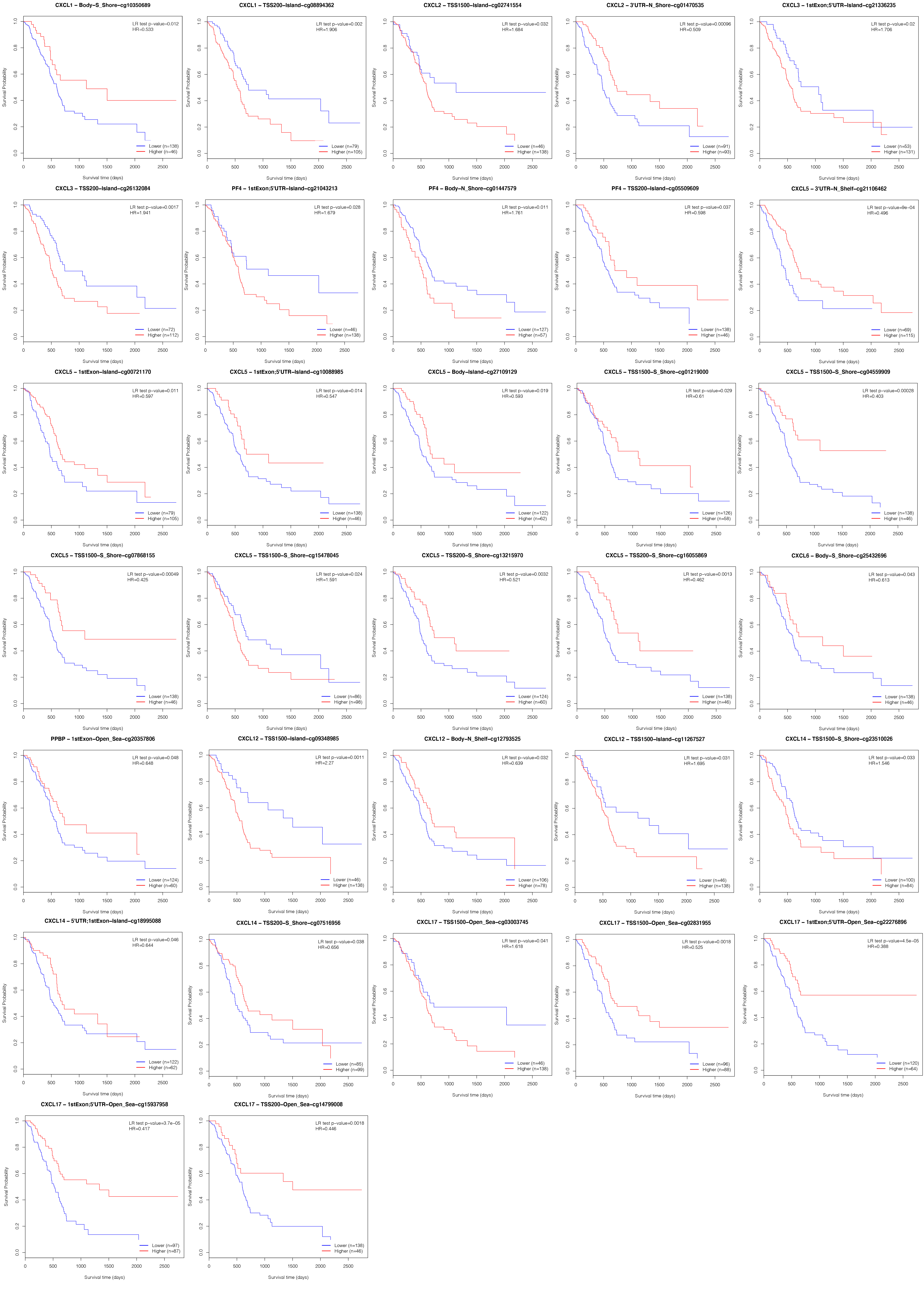

Supplement: Supplementary Figure 7 — Kaplan–Meier curves for visualizing single CpG of CXC Chemokine together with their prognostic value in PAAD (MethSurv). [file Image_7.tif]

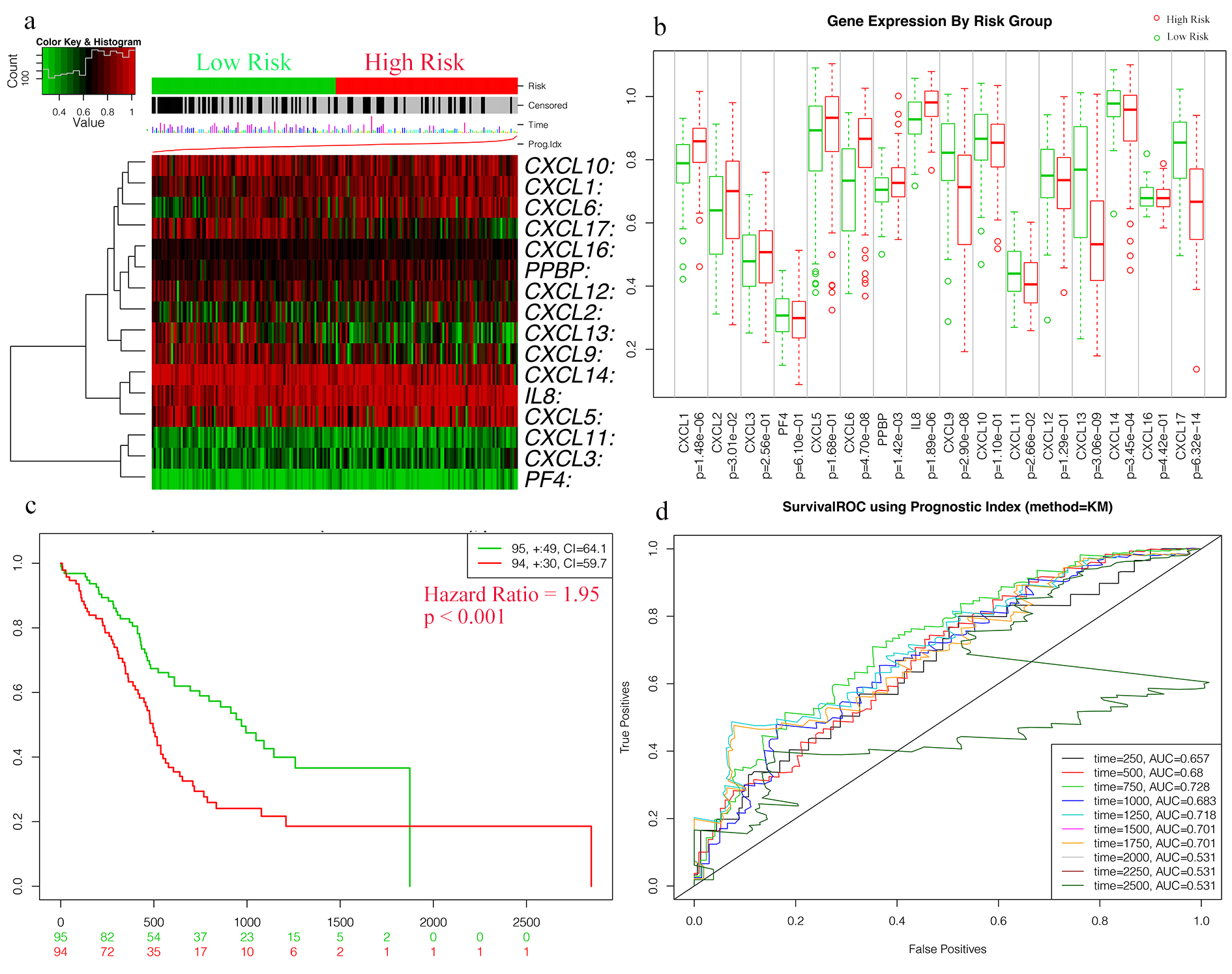

Supplement: Supplementary Figure 8 — The expressions levels and prognostic values of CXC chemokines signature in validation cohort (SurvExpress). (A) The heatmap of CXC chemokines in PAAD patients in high- and low-risk group. (B) The expression levels of CXC chemokines between high- and low-risk groups. (C) Kaplan–Meier curves for survival analysis of CXC chemokines between high- and low-risk groups. (D) The survivalROC curves for survival prediction by the CXC chemokines assessed the accuracy of prognostic model. [file Image_8.tif]

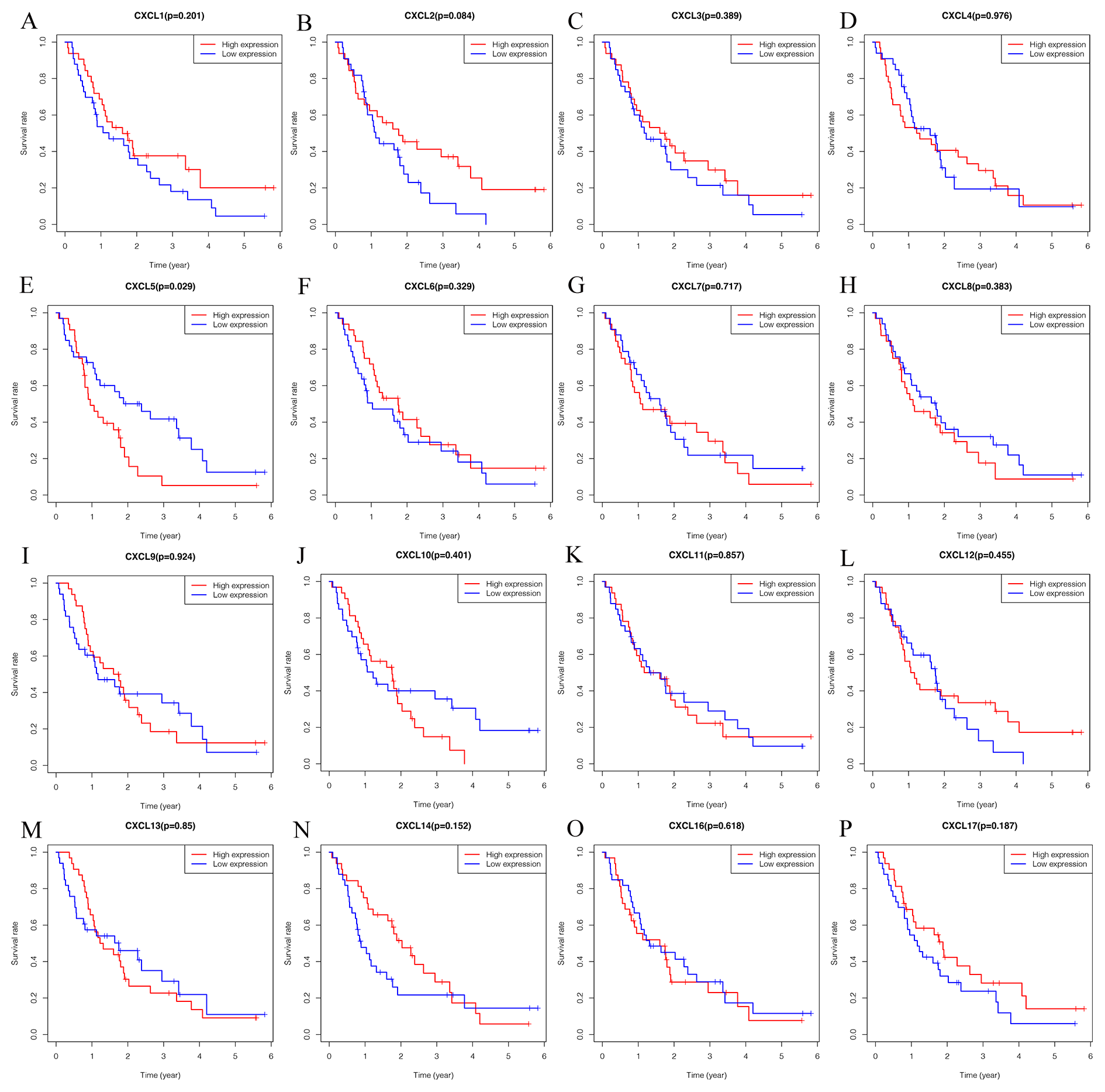

Supplement: Supplementary Figure 9 — External validation of the prognostic value of single CXC chemokine in independent GSE62452 cohort (GEO database). [file Image_9.tif]
