# Supplementary material for: Prognostic Biomarkers and Immunotherapeutic Targets Among CXC Chemokines in Pancreatic Adenocarcinoma
Source: Front Oncol. 2021 Aug 23;11:711402. doi: 10.3389/fonc.2021.711402 (PMC8419473; doi:10.3389/fonc.2021.711402)
Supplement: Supplementary file 10 [file Table_1.docx]

| Genes | Beta | *p -* Value |
| --- | --- | --- |
| CXCL1 | -0.594 | 0.142807039 |
| CXCL2 | 0.133 | 0.663508461 |
| CXCL3 | -0.502 | 0.120853263 |
| CXCL4 | 0.839 | 0.063062504 |
| CXCL5 | 0.426 | 0.015265204 |
| CXCL6 | 0.188 | 0.376985402 |
| CXCL7 | 0.372 | 0.091885664 |
| CXCL8 | 0.488 | 0.120346097 |
| CXCL9 | 0.002 | 0.994028503 |
| CXCL10 | 0.475 | 0.117773332 |
| CXCL13 | -0.137 | 0.309090468 |
| CXCL14 | -0.092 | 0.66399997 |
| CXCL16 | -1.553 | 0.045054878 |
| CXCL17 | 0.386 | 0.008907479 |

**Table S1** The “Beta” of each CXC chemokine (SurvExpress, data from TCGA, Pancreatic adenocarcinoma).

***TCGA*** The Cancer Genome Atlas; ***CXCL*** C-X-C chemokine ligand
