# Supplementary material for: Prognostic Biomarkers and Immunotherapeutic Targets Among CXC Chemokines in Pancreatic Adenocarcinoma
Source: Front Oncol. 2021 Aug 23;11:711402. doi: 10.3389/fonc.2021.711402 (PMC8419473; doi:10.3389/fonc.2021.711402)
Supplement: Supplementary file 11 [file Table_2.docx]

| **GO** | **Category** | **Description** | **Count** | **%** | **Log10(P)** | **Log10(q)** |
| --- | --- | --- | --- | --- | --- | --- |
| R-HSA-380108 | Reactome Gene Sets | Chemokine receptors bind chemokines | 14 | 87.5 | -36.15 | -31.85 |
| GO:0030595 | GO Biological Processes | Leukocyte chemotaxis | 16 | 100 | -33.72 | -29.73 |
| WP3929 | Wiki Pathways | Chemokine signaling pathway | 11 | 68.75 | -21.1 | -18.28 |
| ko04657 | KEGG Pathway | IL-17 signaling pathway | 7 | 43.75 | -13.42 | -10.68 |
| GO:0002690 | GO Biological Processes | Positive regulation of leukocyte chemotaxis | 6 | 37.5 | -11.12 | -8.4 |
| M222 | Canonical Pathways | PID CXCR3 PATHWAY | 5 | 31.25 | -10.55 | -7.84 |
| ko05323 | KEGG Pathway | Rheumatoid arthritis | 5 | 31.25 | -8.9 | -6.29 |
| GO:0098542 | GO Biological Processes | Defense response to other organism | 6 | 37.5 | -6.16 | -3.74 |
| GO:0001817 | GO Biological Processes | Regulation of cytokine production | 3 | 18.75 | -2.02 | 0 |

**Table S2** The pathway and process enrichment analysis of CXC chemokines (Metascape).

***CXCL*** C-X-C chemokine ligand, ***GO*** Gene Ontology, ***KEGG*** Kyoto Encyclopedia of Genes and Genomes
